# Transcriptional plasticity of schizotrophic *Sclerotinia sclerotiorum* responds to symptomatic rapeseed and endophytic wheat hosts

**DOI:** 10.1128/spectrum.02612-23

**Published:** 2023-10-31

**Authors:** Binnian Tian, Ziyang Chen, Yang Yu, Yuheng Yang, Anfei Fang, Chaowei Bi, Zheng Qu, Yanping Fu, Mirza Abid Mehmood, Changyong Zhou, Daohong Jiang

**Affiliations:** 1 College of Plant Protection, Southwest University, Chongqing, China; 2 Key Laboratory of Agricultural Biosafety and Green Production of Upper Yangtze River (Ministry of Education), Southwest University, Chongqing, China; 3 State Key Laboratory of Agricultural Microbiology, Huazhong Agricultural University, Wuhan, China; 4 The Provincial Key Lab of Plant Pathology of Hubei Province, Huazhong Agricultural University, Wuhan, China; 5 Plant Pathology, Institute of Plant Protection, Muhammad Nawaz Shareef University of Agriculture, Multan, Pakistan; Instituto de Ecología, Pátzcuaro, Michoacán, Mexico

**Keywords:** schizotrophic fungi, Sclerotinia sclerotiorum, transcriptional plasticity, symptomatic host, endophytic host

## Abstract

*Sclerotinia sclerotiorum*, a widespread pathogen of dicotyledons, is also a reciprocal endophytic fungus in monocot plants. However, it is unclear how it responds to two distinct plants. Comparative transcriptomic analyses uncovered a network rewiring, with 14.1% up-differentially expressed genes overlapping upon colonizing the endophytic host wheat and a symptomatic host rapeseed. The divergent transcript of genes originating from host-specific induction is central to infection and colonization, including genes related to appressorium, oxalic acid, carbohydrate-active enzymes, and effector-like proteins. We confirmed that appressorium is required for *S. sclerotiorum* during colonization in symptomatic hosts but not in endophytic wheat via the observation of the infection process of *ΔSs-caf1*, a compound appressorium formation-defective mutant with hypovirulence to symptomatic hosts. *SS1G_10617* and *SS1G_13809* are components of the starch degradation pathways. *ΔSS1G_10617* displayed lower virulence on symptomatic host plants and abnormal endophytic growth on wheat, and *ΔSS1G_13809* only showed an abnormal symbiotic relationship with wheat, implying that starch may be the major carbon source for *S. sclerotiorum* in the symbiosis with wheat. Our results herein suggest that *S. sclerotiorum* modulates biphasic colonization via a divergent transcriptional landscape. This study contributes to a better understanding of schizotrophic fungi and provides new clues for cultivating disease-resistant varieties and adjusting reasonable farming strategies.

**IMPORTANCE:**

The broad host range of fungi with differential fungal responses leads to either a pathogenic or an endophytic lifestyle in various host plants. Yet, the molecular basis of schizotrophic fungal responses to different plant hosts remains unexplored. Here, we observed a general increase in the gene expression of *S. sclerotiorum* associated with pathogenicity in symptomatic rapeseed, including small protein secretion, appressorial formation, and oxalic acid toxin production. Conversely, in wheat, many carbohydrate metabolism and transport-associated genes were induced, indicating a general increase in processes associated with carbohydrate acquisition. Appressorium is required for *S. sclerotiorum* during colonization in symptomatic hosts but not in endophytic wheat. These findings provide new clues for understanding schizotrophic fungi, fungal evolution, and the emergence pathways of new plant diseases.

## INTRODUCTION

Plants and microbes have developed associations that range from mutualistic to parasitic in natural ecosystems. These interactions occur when fungi live in plants and cause damage or exert beneficial effects for their host (1). Both the manipulation of the plant immune system and the alteration of host development processes contribute to the multiple symptoms caused by fungi in plants during colonization (2 – 4). Transcriptomic and genomic analyses indicate that fungi have a delicate and complex infection program, although studies surveying single-trait interactions have emphasized key processes and pathways necessary for the pathogenesis of fungi in plants (5 – 8). According to transcriptomics during fungal colonization, infection programs vary depending on the lifestyle of the fungus, which suggests that the induced transcriptional landscapes diverge in the interaction of biotrophic, hemibiotrophic, and/or necrotrophic pathogens (9). However, most studies have focused on finding potential regulators based on the interaction between the fungus and a particular host. Although clearly instructive, the findings of these studies have left significant gaps in our understanding of how individual fungi infect multiple hosts.

Plant fungal pathogens often have broad host ranges, emphasizing their potential to cause huge economic losses in agricultural systems. Most plant pathogens have only narrow host ranges, while several fungi have wide host ranges involved in asymptomatic host colonization and development (10). The hemibiotrophic pathogens *Colletotrichum* spp. can also endophytically grow in endophytic hosts and exert beneficial effects on them (11); *Verticillium dahliae* is a destructive pathogen known to attack more than 400 different hosts, and at the same time, it also has a broad endophytic host range (12). In addition, *Fusarium virguliforme*, which causes sudden death syndrome in soybeans, also acts as an endophyte in a variety of asymptomatic host plants (13). This demonstrates that individual fungi can manipulate their genetic expression network to enable the colonization of hosts with specific pathogenic or endophytic effects. Furthermore, Baetsen-Young et al. uncovered that *F. virguliforme* reprograms its transcriptome to adopt a necrotrophic or biotrophic lifestyle rather than expressing unique transcripts in symptomatic host soybean or endophytic host maize. Hence, studying fungal species that have both endophytic and symptomatic host phenotypes offers an opportunity to comprehend the transcriptional reprogramming necessary for colonizing hosts. In addition, it was discovered that potential carbohydrate-active enzymes, necrosis-inducing effectors, and Zn(II)-Cys6 transcription factors were major factors in the divergence of *F. virguliforme* infection and colonization (9). However, whether the major carbon source in the symptomatic and asymptomatic hosts differs is still unknown.

*Sclerotinia sclerotiorum* is a typical necrotrophic fungus with a broad range of hosts, including numerous economically important cultivated species like rapeseed and soybean (14, 15). It is also one of the most destructive agricultural diseases and causes large annual global economic losses due to its adaptations for long-term survival and pathogenicity on numerous hosts (10). The virulence-related secretory effector proteins, oxalic acid (OA), plant cell wall-degrading enzymes (PCWDEs), and the appressorium enable it to successfully colonize its symptomatic hosts and modulate host defense signaling (16). Cereal crops such as wheat and rice are usually considered nonhosts of *S. sclerotiorum* and are widely used in rotation with susceptible host crops (17, 18). Previously, we proved that *S. sclerotiorum* can grow endophytically in wheat, which promotes wheat growth and offers protection against two major fungal diseases. We also termed it schizotrophism, the host-dependent fungus tropism, destructively pathogenic or mutualistically endophytic (19). However, the schizotrophic molecular determinants between *S. sclerotiorum* and the symptomatic host rapeseed and endophytic host wheat are largely unknown.

Digital RNA sequencing (RNA-seq), also known as unique molecular identifier (UMI) RNA-seq, was developed to effectively solve the sequence-dependent bias and the impreciseness of polymerase chain reaction (PCR) amplification by tagging each cDNA molecule with a UMI before library construction. It has been widely used to identify genetic signatures associated with pathogenicity and compatible host-pathogen interactions (20, 21). Here, we systematically compared the host-pathogen transcriptomic interface during symptomatic and endophytic colonization using UMI RNA-seq. Highlighting the divergence of genetic signaling pathways that underpin fungal lifestyles, we investigated (1) the phenotypes and mycelial morphology at the early stages of colonization of *S. sclerotiorum* on symptomatic rapeseed host and endophytic wheat host (2); the potential conservation or distinction between endophytic versus symptomatic fungal transcriptomes (3); how transcriptional responses of *S. sclerotiorum* colonizing rapeseed or wheat are regulated; and (4) the function of two glucoamylase genes in regulating the establishment of symbiotic relationships between *S. sclerotiorum* and endophytic wheat host, and in modulating the pathogenicity of *S. sclerotiorum* on symptomatic hosts.

## RESULTS

### Colonization of *S. sclerotiorum* on the endophytic wheat host root was not dependent on the appressorium

**Fig 1:**
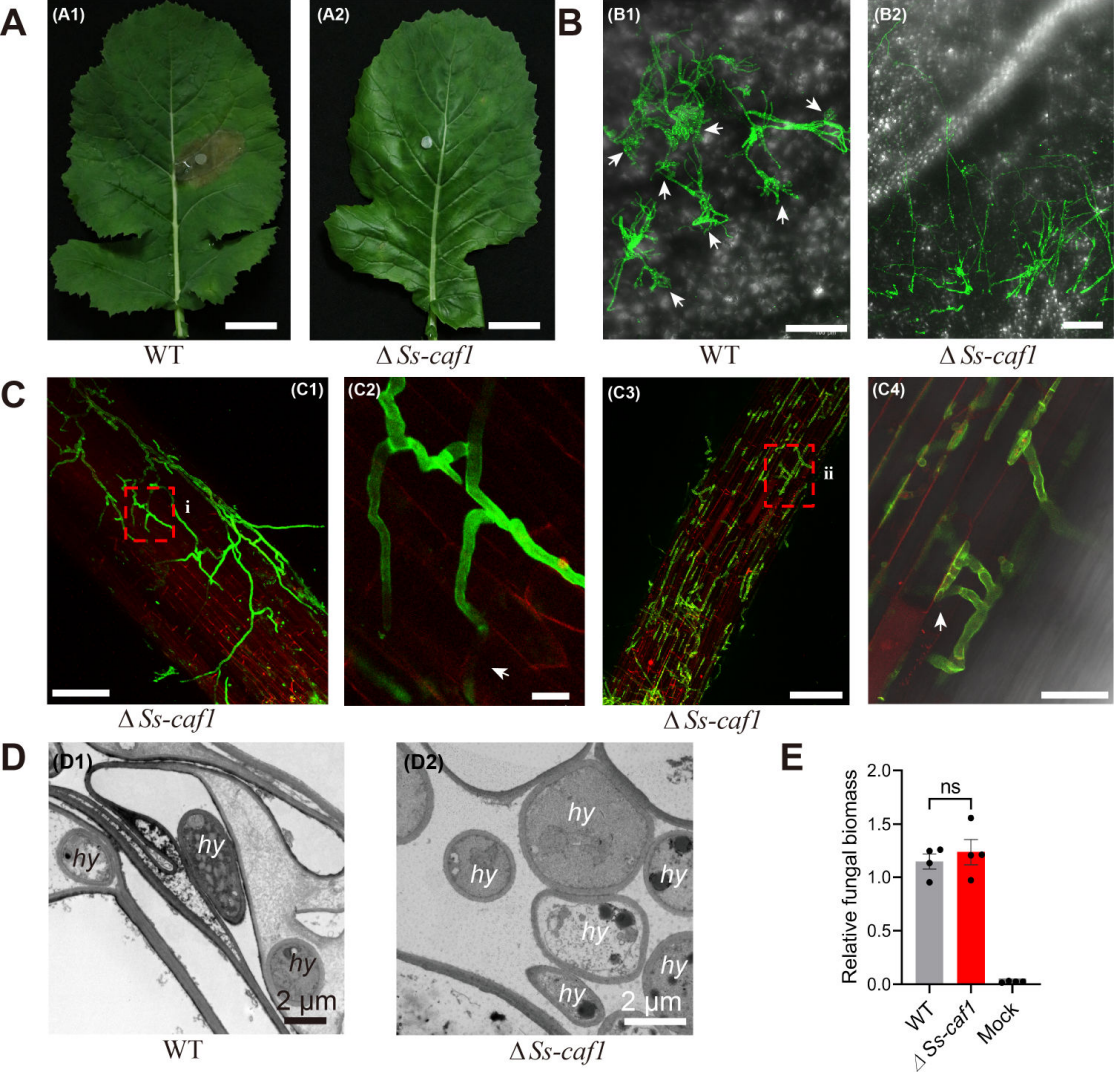
Appressorium is not required for *Sclerotinia sclerotiorum* colonization in wheat roots. (**A**) Virulence assays of *S. sclerotiorum* WT strain (A1) and *ΔSs-Caf1* (A2) on the detached rapeseed leaves. Photographs were taken at 2 dpi. Scale bars, 2 cm. (**B**) Representative image of the *S. sclerotiorum* WT strain (B1) and *ΔSs-Caf1* strain (B2) mycelial morphology growing on the detached rapeseed leaves at 10 and 2 dpi, respectively. White arrows indicate appressoria of *S. sclerotiorum*; hyphae were stained with WGA. Scale bars for B1 and B2 are 100 and 50 µm, respectively. (**C**) Confocal microscope images of *ΔSs-Caf1* hyphae colonizing wheat root epidermal cells; (C1 and C3) seedling wheat root samples were taken at 2 and 12 dpi, respectively. (C2) Enlargements of the boxed region (i) in panel C1; *ΔSs-Caf1* hyphae penetrating a wheat root epidermal cell (arrows); (C4) Enlargements of the boxed region (ii) in panel C3; white arrows indicate *ΔSs-Caf1* hyphae in a wheat epidermal root cell; hyphae were stained with WGA and wheat plant cell-wall apoplastic space was stained with PI. Scale bars for panels C1, C2, C3, and C4 are 100, 20, 100, and 40 µm. (**D**) Hyphae of WT and *ΔSs-Caf1* in the wheat roots visualized with TEM; hy, fungal hyphae. Scale bars, 2 µm. (**E**) The biomass of *S. sclerotiorum* WT and *ΔSs-Caf1* in roots of wheat measured by qPCR using total DNA extracted at 12 dpi. Wheat roots inoculated with water were used as a mock. Error bars indicate SD; *n* = 4 biological replicates.

Given the lack of understanding of how *S. sclerotiorum* interacts with the symptomatic host (rapeseed) and endophytic host (wheat), a systematic investigation was performed on the *S. sclerotiorum*-host infection phenotypes, mycelial morphology and structure, and transcriptomic interface during symptomatic and endophytic colonization. Rapeseed stems and wheat roots were observed 2 days after inoculation with *S. sclerotiorum* using confocal microscopy, and RNA samples were collected for UMI RNA-sequencing analysis. We found that stems showed a necrotic lesion of *Sclerotinia* stem rot (SSR) at 2 days post inoculation (dpi) (Fig. S1A). Conversely, there was no striking evidence of root necrosis on the endophytic host wheat (Fig. S1C). Many appressoria were produced during the early infection stages of *S. sclerotiorum* in rapeseed stalks, interestingly, but not in endophytic host wheat (Fig. S1B and D). To investigate whether appressoria are necessary for the colonization of *S. sclerotiorum* on wheat, *ΔSs-caf1*, a compound appressorium formation-defective mutant with hypovirulence to rapeseed (Fig. 1A and B) (22), was inoculated on the wheat seedling roots. We found that *ΔSs-caf1* hyphae could directly enter and further grow inside the root epidermal cells of wheat by confocal microscopy at 2 (Fig. 1C1 and C2) and 12 dpi (Fig. 1C3 and C4). Furthermore, transmission electron microscopy (TEM) also revealed that *ΔSs-caf1* hyphae could be observed both in root cells and intercellular space (Fig. 1D). To further evaluate the colonization ability of *ΔSs-caf1* on wheat, the relative biomass of *ΔSs-caf1* and the wild-type (WT) strain bwas detected by quantitative polymerase chain reaction (qPCR) in wheat roots at 12 dpi. The qPCR results showed that the colonization ability of *ΔSs-caf1* was no different compared to that of the wildtype (Fig. 1E). Thus, these results suggest that appressorium is not required for *S. sclerotiorum* colonization in endophytic wheat roots.

### Overall assessment of host-induced gene expression profiles of *S. sclerotiorum*

A comparative transcriptomic-based approach was performed to determine whether the colonization profile of *S. sclerotiorum* differed in a manner consistent with the host phenotype and mycelial morphology at 2 dpi. In fungal samples from the rapeseed (SR) and wheat (SW) groups, 237.2 and 273.1 million total reads were obtained, of which approximately 80.43% and 45.40% could be aligned to the *S. sclerotiorum* genome, respectively. For the *S. sclerotiorum* control (Ss) group (incubated on Murashige and Skoog [MS] medium), there were 284.4 million total reads and about 95.15% of reads could be aligned to the *S. sclerotiorum* genome. To determine whether *S. sclerotiorum* treatments were globally distinct from one another, a principal component analysis and sample reads per kilobase per million mapped reads (RPKM) correlation analysis were performed and fungal responses on different hosts did cluster distinctly from each other (Fig. S2A and S2B). The grouping of samples by hosts suggests that the symptomatic or endophytic host plant-fungal interactions greatly shaped the *S. sclerotiorum* gene expression pattern.

**Fig 2:**
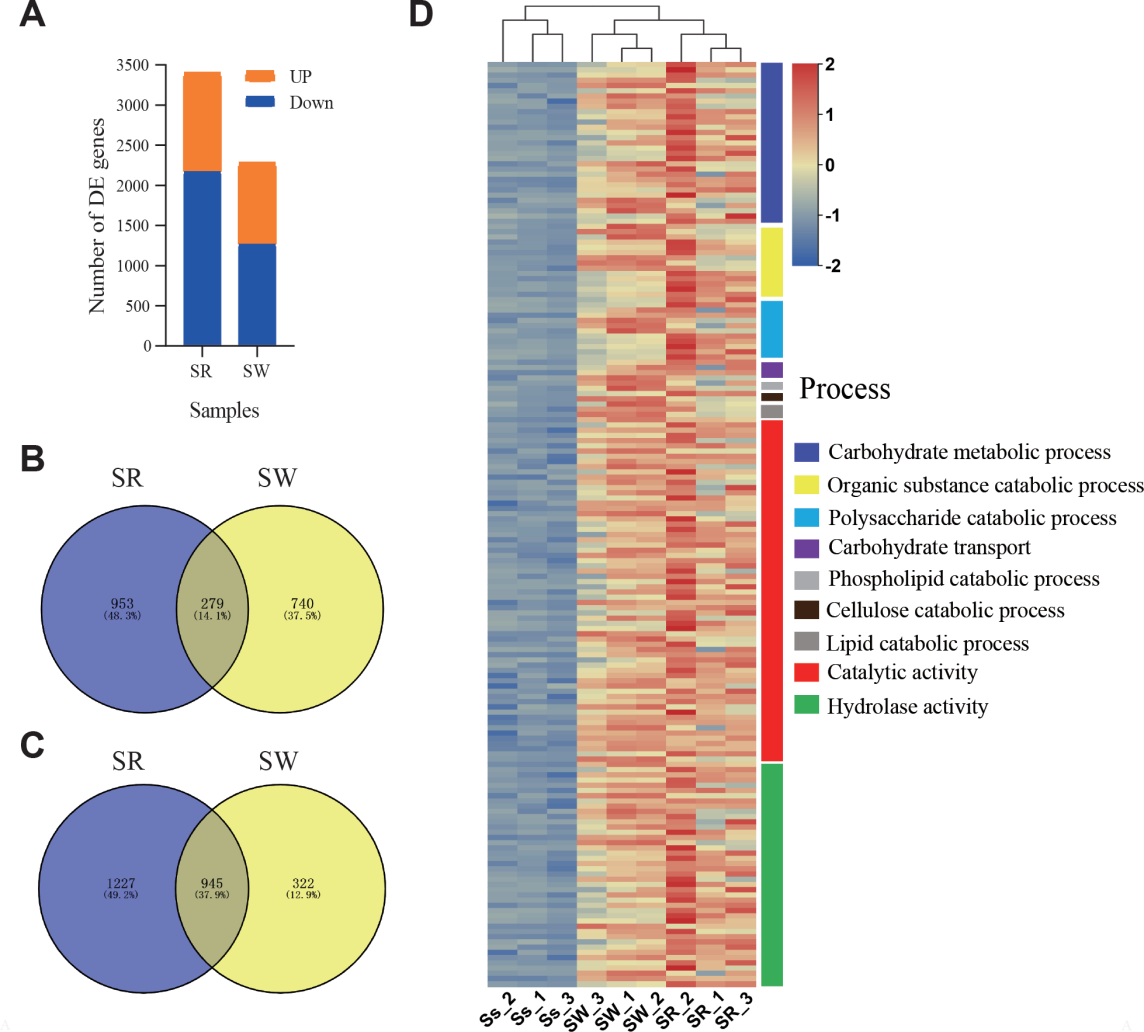
Overall assessment of the host-induced gene expression profiles in *Sclerotinia sclerotiorum*. (**A**) The number of differentially expressed (|log_2_FC| > 1, FDR < 0.05) *S. sclerotiorum* genes in rapeseed stem or wheat root hosts versus hyphae of *S. sclerotiorum* on MS. (**B** and **C**) Venn diagram of the upregulated (**B**) and downregulated (**C**) *S. sclerotiorum* genes in rapeseed stem and wheat root samples. (**D**) Heat maps of significant enrichment of gene ontology categories of commonly upregulated *S. sclerotiorum* genes (|log_2_FC| > 1, FDR < 0.05) during the colonization of rapeseed and wheat (*n* = 177).

To discover *S. sclerotiorum* genes induced by host interaction, we next compared gene expression patterns of *S. sclerotiorum* on rapeseed or wheat with growth on MS media. A total of 3,404 and 2,286 *S*. *sclerotiorum* genes were found to be differentially expressed (|log_2_ ratio| ≥ 1 and FDR < 0.05), of which 1,232 (36.19%) and 1,019 (44.58%) differentially expressed genes (DEGs) were upregulated, respectively (Fig. 2A; Fig. S2C and S2D). Furthermore, 279 (14.10%) and 945 (37.89%) genes were commonly upregulated and downregulated on rapeseed and wheat samples, respectively (Fig. 2B and C).

Gene ontology (GO) enrichment analysis was performed to explore the function of host-induced common upregulated genes in *S. sclerotiorum*. A total of 37 biological process categories and 11 molecular function categories were enriched and consistently upregulated on both rapeseed and wheat (Fig. 2D; Table S1). The overall expression patterns on both hosts were similar for carbohydrate metabolic process, carbohydrate transport, polysaccharide, organic substance, lipid catabolic process, phospholipid catabolic process, carbohydrate transport, catabolic activity, and hydrolase activity, which is not surprising, as these processes are critical for fungal growth (23 – 27), secondary metabolite production (28, 29), and fungal nutrient acquisition and growth (25).

For the 945 host-induced common downregulated genes, there were 23 biological process categories and 36 molecular function categories were enriched (Table S2) and 6 significantly enriched GO terms were related to the “transport,” namely, “transmembrane transport,” “phospholipid transport,” “anion transport,” “lipid transport,” “organophosphate ester transport,” and “organic anion transport.” Meanwhile, “catabolic activity,” “hydrolase activity,” “lipid catabolic process,” “ion antiporter activity,” “proton antiporter activity,” and “cation antiporter activity” were also the enriched GO terms. These GO terms might be related to the growth of *S. sclerotiorum* (25, 27).

### Symptomatic host-induced gene expression profiles in *S. sclerotiorum*

**Fig 3:**
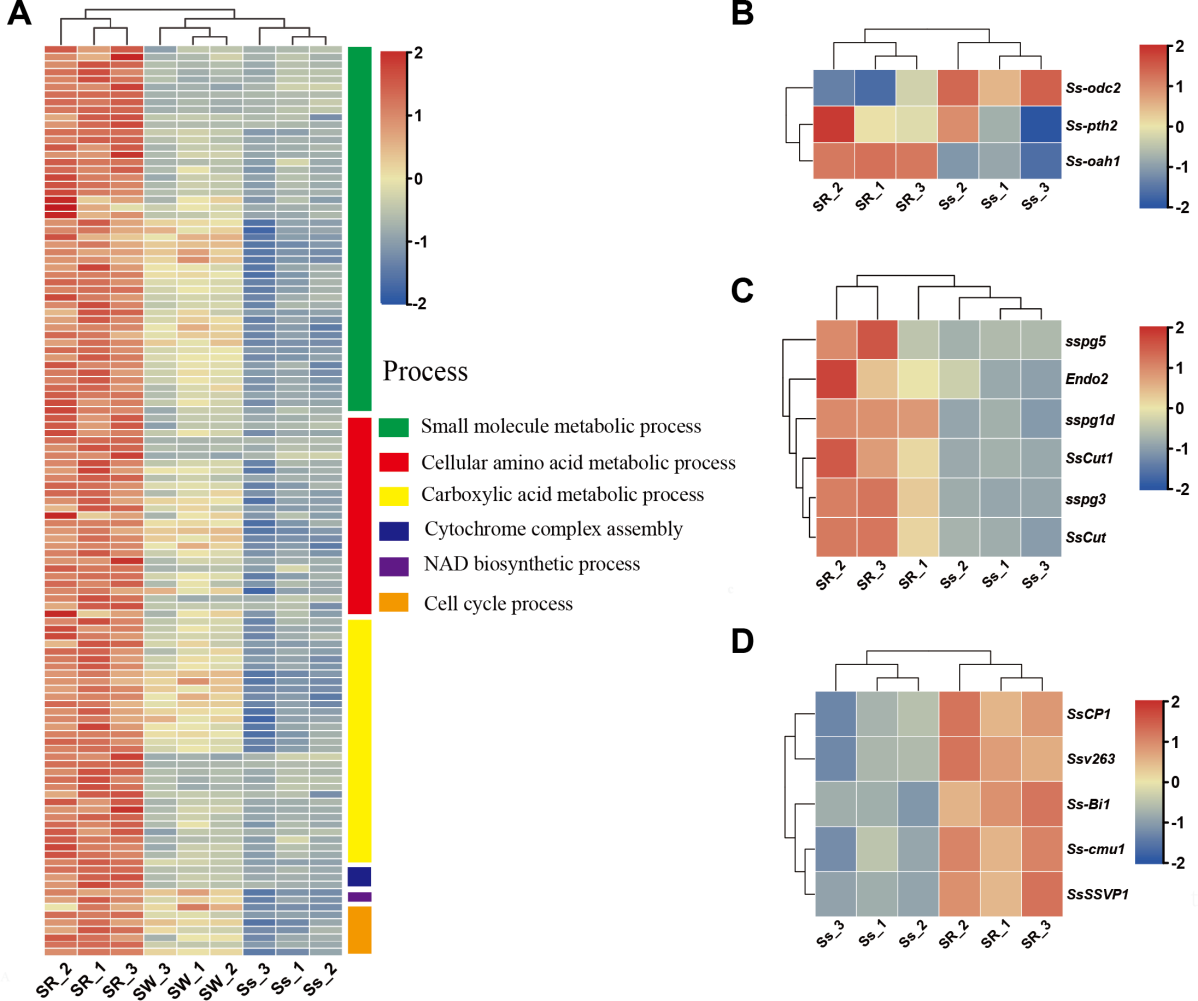
Symptomatic host rapeseed-uniquely induced gene expression profiles in *Sclerotinia sclerotiorum*. (**A**) Heat maps of significant enrichment of gene ontology categories of uniquely upregulated *S. sclerotiorum* genes (|log_2_FC| > 1, FDR < 0.05) during the colonization of rapeseed stem (*n* = 114). (**B**) Gene expression profiles of *S. sclerotiorum* key genes of oxalic acid metabolism and regulation during *S. sclerotiorum* colonization of rapeseed stem (*n* = 3). (**C**) Expression profiles of *S. sclerotiorum* identified PCWDEs during the colonization of rapeseed stem (*n* = 6). (**D**) Expression profiles of *S. sclerotiorum* identified SP encoding genes during the colonization of rapeseed stem (*n* = 5).

Previous work comparing the infection profiles of *F. virguliforme* on a symptomatic host soybean and endophytic host maize revealed host-specific variation of *F. virguliforme* (9). To determine whether this is also the case in *S. sclerotiorum*, we directly compared *S. sclerotiorum* gene expression patterns from the symptomatic and endophytic hosts. There were 10,247 genes (69.65%) in the *S. sclerotiorum* transcriptome, which were not differentially regulated in cross-species colonization. Of the proportion of genes that were differentially induced, 21.34% (953 genes) and 27.47% (1,227 genes) were uniquely upregulated and downregulated during rapeseed stem infection, respectively (Fig. 2B and C). For the 953 upregulated genes, more than 60 GO terms were significantly enriched, including small molecule biosynthesis, cellular amino acid metabolic process, carboxylic acid metabolic process, cytochrome complex assembly, NAD metabolic process, and cell cycle process (Fig. 3A; Table S3).

The compound appressorium of *S. sclerotiorum*, a multicellular, melanin-rich hyphal penetration structure, becomes extensively branched, hook, and bifurcate when contacting with the host and is proved to be involved in pathogenicity (22, 30). Autophagy, cell cycle process, and cytochrome complex assembly in plant pathogenic fungal hyphae support the association with the formation of infection structures like appressoria (31 – 33). NAD metabolic process has been previously associated with hyphal differentiation initiation for infection structures (34, 35). These three GO terms (cell cycle process, cytochrome complex assembly, and NAD metabolic process) were significantly enriched in the genes upregulated in our *S. sclerotiorum*-inoculated rapeseed stems (Fig. 3A). Noteworthy, three *S*. *sclerotiorum* DEGs (*SS1G_05459*, *SS1G_06363*, and *SS1G_10707*) associated with autophagy were downregulated in our *S. sclerotiorum*-inoculated rapeseed stems. This is consistent with what we observed in the development of appressoria-like structures during the early infection stages of *S. sclerotiorum* in rapeseed plants.

OA is a multifunctional molecule with a range of functions in *S. sclerotiorum*, such as the reduction of the pH of host tissue, chelation of calcium to weaken the host cell wall structure, reduction of host calcium toxicity, and suppression of host defense responses (36). The oxaloacetate acetylhydrolase gene *Ss-oah1* and peroxysomal carnitine acetyltransferase gene *Ss-pth2* were upregulated, and the oxalate decarboxylase enzyme gene *Ssodc2* was downregulated in this study (Fig. 3B), which is consistent with the function of OA in the pathogenesis of *S. sclerotiorum*. Furthermore, PCWDEs are well-known important factors in *S. sclerotiorum* infection of the symptomatic host, *sspg5*, *Endo2*, *sspg1d*, *SsCut1*, *sspg3*, and *SsCut* were upregulated during rapeseed stem colonization in this study (Fig. 3C; Fig. S5). The small molecule biosynthesis processes are potentially associated with the function of necrotrophic effectors (37, 38). The genome of *S. sclerotiorum* contains 486 genes encoding secretory proteins (SPs) expressed *in planta* according to the prediction (39). In this study, 68 out of 135 upregulated SP encoding genes were unique during rapeseed stem colonization*,* including pathogenesis-related *SsCP1*, *Ssv263*, *Ss-Bi1*, *Ss-cmu1,* and *SsSSVP1* (Fig. 3D; Fig. S3B) (16, 40 – 43).

Furthermore, the cellular amino acid metabolic process may be associated with the virulence of *S. sclerotiorum* (25), and the carboxylic acid metabolic process is a critical process for secondary metabolite production (29). Based on these upregulated genes, “valine, leucine, and isoleucine degradation,” “aminoacyl-tRNA biosynthesis,” “pentose and glucuronate interconversions,” and “glycosylphosphatidylinositol (GPI)-anchor biosynthesis,” Kyoto Encyclopedia of Genes and Genomes (KEGG) pathways were significantly enriched (Fig. S4B). Noteworthily, “glycosylphosphatidylinositol-anchor biosynthesis” and “pentose and glucuronate interconversions” were related to fungal virulence (44 – 47). Taken together, these analyses suggest that *S. sclerotiorum* manipulates the rapeseed host responses via appressorium, oxalic acid, SPs, and PCWDEs and further facilitates its infection to cause necrotic lesions.

### Endophytic host-induced gene expression profiles in *S. sclerotiorum*

**Fig 4:**
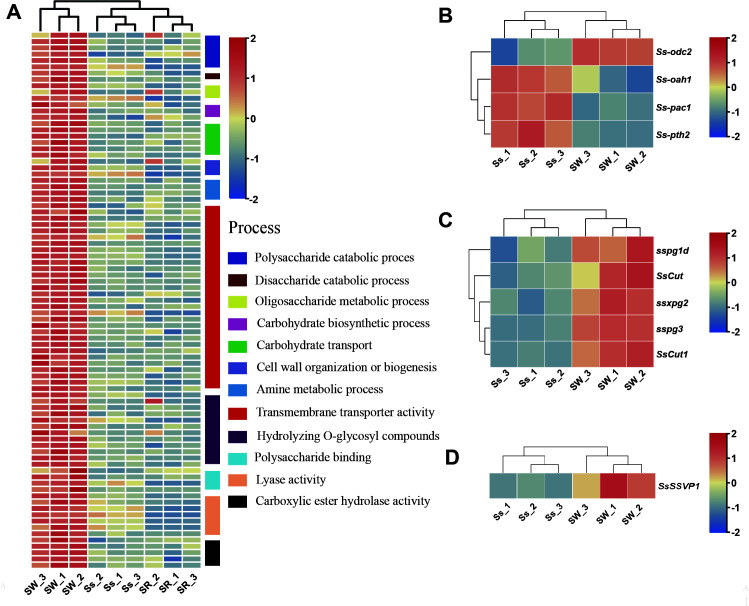
Endophytic host wheat-uniquely induced gene expression profiles in *Sclerotinia sclerotiorum*. (**A**) Heat maps of significant enrichment of gene ontology categories of uniquely upregulated *S. sclerotiorum* genes (|log_2_FC| > 1, FDR < 0.05) during colonization of wheat root (*n* = 86). (**B**) Gene expression profiles of *S. sclerotiorum* key genes of oxalic acid metabolism and regulation during *S. sclerotiorum* colonization of wheat root (*n* = 4). (**C**) Expression profiles of *S. sclerotiorum* identified PCWDEs during the colonization of wheat root (*n* = 5). (**D**) Expression profiles of *S. sclerotiorum* identified SP encoding genes during the colonization of wheat root (*n* = 1).

A total of 740 (16.57%) and 322 (7.21%) DEGs were uniquely upregulated and downregulated in *S. sclerotiorum* during wheat root colonization, respectively. GO enrichment analysis of uniquely upregulated genes showed that many processes related to carbohydrate metabolism, including carbohydrate biosynthetic process, oligosaccharide metabolic process, polysaccharide and disaccharide catabolic process, cell wall organization or biogenesis, and carbohydrate transport were enriched when *S. sclerotiorum* colonized wheat roots at 2 dpi. Furthermore, transmembrane transporter activity, hydrolyzing O-glycosyl compounds, polysaccharide binding, amine metabolism process, lyase activity, and carboxylic ester hydrolase activity were also significantly enriched (Fig. 4A; Table S4). The KEGG enrichment analysis showed similar results to the GO enrichment analysis that “starch and sucrose metabolism,” “amino sugar and nucleotide sugar metabolism,” and “thiamine metabolism and methane metabolism” were enriched (Fig. S4A). Twenty-one upregulated (21/55) and three downregulated (3/55) *S. sclerotiorum* DEGs were enriched in the “starch and sucrose metabolism” pathway; noteworthy, seven *S*. *sclerotiorum* DEGs (*SS1G_10617*, *SS1G_07184*, *SS1G_01493*, *SS1G_06037*, *SS1G_09251*, *SS1G_01229*, and *SS1G_07847*) encode alpha-amylase or glucoamylase. These results suggested that *S. sclerotiorum* may rely on starch as the main carbon source during the colonization of endophytic wheat.

When colonizing wheat roots, as to OA, only *Ssodc2* was upregulated in *S. sclerotiorum*, interestingly, *Ss-oah1*, *Ss-pth2,* and positive transcription factor gene *Ss-pac1* were downregulated (Fig. 4B). This finding suggested that OA might be not so important for the colonization of *S. sclerotiorum* in endophytic host wheat as in rapeseed. PCWDEs are key factors during root colonization for both endophytic and pathogenic fungi with hemibiotrophic lifestyles (48). Noteworthy, the expression of PCWDE-related genes *sspg1d, SsCut*, *ssxpg2*, *sspg3,* and *SsCut1* was also upregulated during wheat root colonization, which was the same as that in the symptomatic host rapeseed colonization (Fig. 4C; Fig. S5). This result indicated that PCWDEs also play important roles in the establishment of the symbiotic relationship between *S. sclerotiorum* and endophytic host wheat. Additionally, 67 SP encoding genes, including *SsSSVP1*, were also upregulated during wheat root colonization and consistent with the colonization of rapeseed stem in *S. sclerotiorum* (Fig. 4D; Fig. S3A). These SPs might modulate the infection of *S. sclerotiorum* and manipulate the plant immune system on both symptomatic host rapeseed and endophytic host wheat. Noteworthily, 87 out of the 154 upregulated SP encoding genes were unique during wheat root colonization in *S. sclerotiorum* (Fig. S3C; Table S5). Thus, these upregulated SP encoding genes might help *S. sclerotiorum* eliminate the adverse effects of the activated defense response of wheat and further promote the establishment of symbiosis between *S. sclerotiorum* and the wheat plant.

### *SS1G_10617* and *SS1G_13809* contribute to the colonization or infection of the asymptomatic and symptomatic hosts

**Fig 5:**
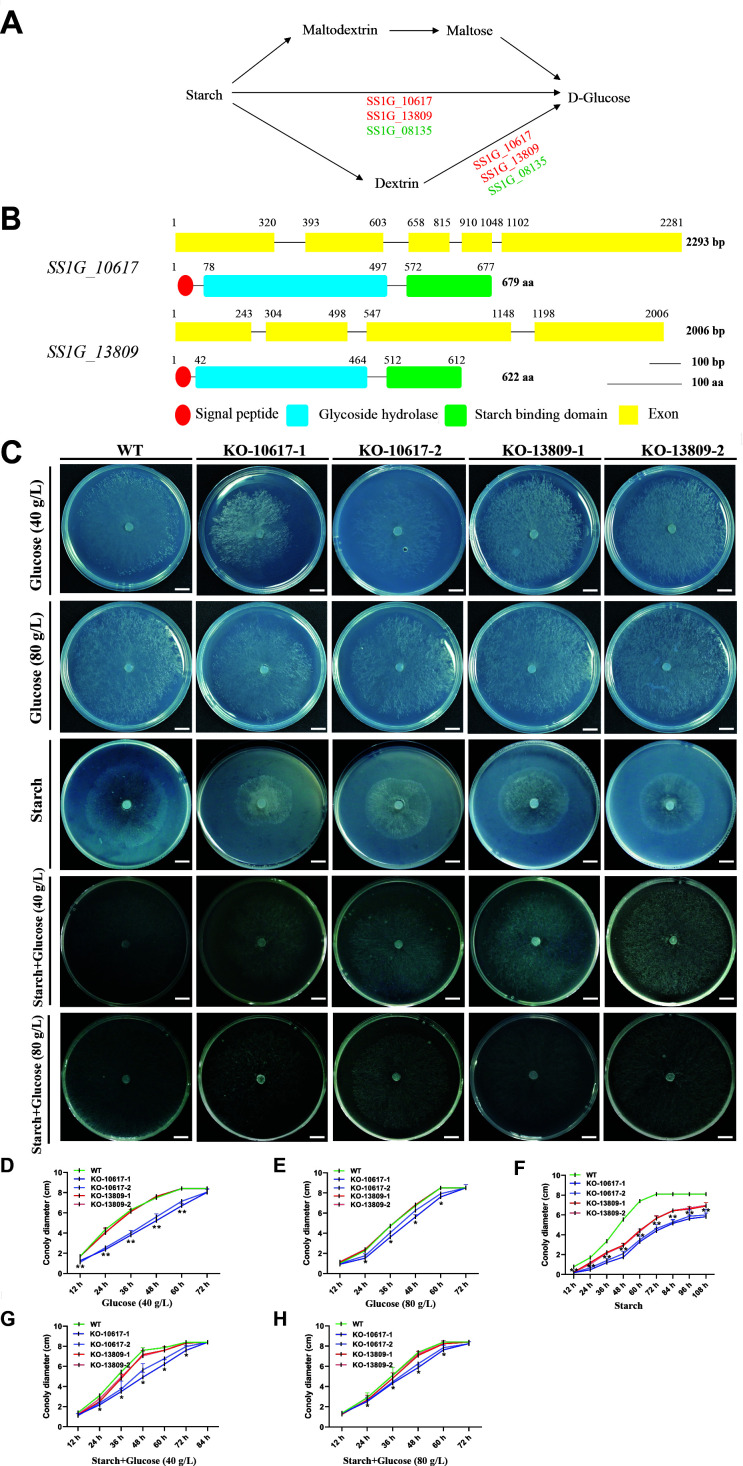
Characterizations of *SS1G_10617* and *SS1G_13809* and mutants colony morphologies. (**A**) Schematic view of the degradation pathways of starch to glucose in *S. sclerotiorum*. (**B**) Conserved domains of SS1G_10617 and SS1G_13809 proteins in *S. sclerotiorum* are shown in proportion to the length of the nucleotide and amino acid sequences. (**C**) Colony morphologies of *SS1G_10617* mutants (KO-10617-1 and KO-10617-2), *SS1G_13809* mutants (KO-13809-1 and KO-13809-2), and WT strain at 20°C for 2 days on the glucose plate (40 and 80 g/L), the starch plate and starch media with glucose (40 and 80 g/L). Scale bar, 1 cm. (**D–H**) The colony diameters of WT, *SS1G_10617* mutants, and *SS1G_13809* mutants on the glucose plate (40 and 80 g/L), the starch plate, and starch media with glucose (glucose 40 or 80 g/L). Error bars indicate SD; *n* = 4 biological replicates. **P < 0.01 and *P < 0.05 in the variance analysis.

**Fig 6:**
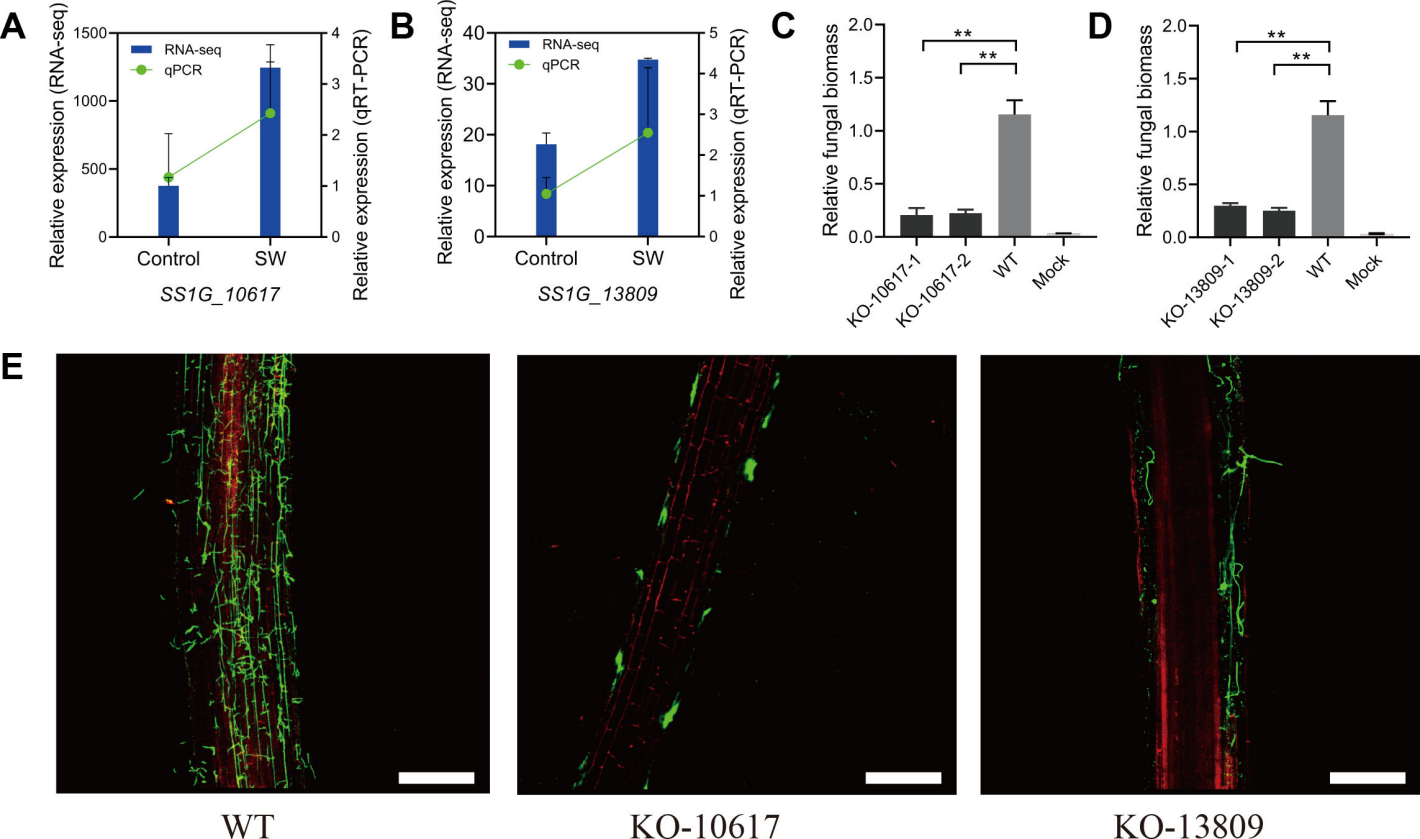
*SS1G_10617* and *SS1G_13809* contribute to the symbiosis between *Sclerotinia sclerotiorum* and wheat. Expression levels of *S. sclerotiorum SS1G_10617* (**A**) and *SS1G_13809* (**B**) in wheat roots at 2 dpi; the *S. sclerotiorum* hyphae on half-strength MS agarose medium for 2 days were used as control. (**C and D**) *S. sclerotiorum* biomass in roots of wheat inoculated with the KO-10617 and KO-13809 strains measured by qPCR using total DNA extracted at 12 dpi. *S. sclerotiorum* DNA was calculated using the threshold cycle (*C*_*t*_) method, normalized to the wheat *TaEF-1a* gene, and expressed relative to that in roots inoculated with WT strain. Wheat plant roots 12 days post-inoculation with water as mocks. Error bars indicate SD; *n* = 4 biological replicates. **P < 0.01. The experiment was performed three times with similar results. (**E**) Confocal microscopy images showing wheat roots inoculated with the *S. sclerotiorum*-WT strain, KO-10617, or KO-13809 at 12 dpi. Hyphae of *S. sclerotiorum* were stained with WGA, and wheat plant cell-wall apoplastic space was stained with PI. Scale bars, 0.25 mm.

Go enrichment analysis suggested that starch might be an important nutrient for *S. sclerotiorum* in the symbiosis between *S. sclerotiorum* and wheat. The degradation of starch to glucose in *S. sclerotiorum* relies mainly on three genes with glycohydrolase domain, *SS1G_10617*, *SS1G_13809*, and *SS1G_08135* (Fig. 5A). *SS1G_10617* and *SS1G_13809* were upregulated at 2 dpi during the colonization of wheat root, but *SS1G_08135* was downregulated (Fig. 6A and B). These results suggested that *SS1G_10617* and *SS1G_13809* likely played an important role during the colonization of wheat plants. SS1G_13809 and SS1G_10617 in *S. sclerotiorum* contain signal peptides in the N terminus, a glycoside hydrolase domain of approximately 420 amino acids, and a 100-amino-acid starch binding domain in the C terminus (Fig. 5B). Phylogenetic analysis revealed that proteins encoded by *SS1G_10617* and *SS1G_13809* are widely present in various kinds of filamentous fungi. Noteworthily, SS1G_10617 is conserved in symbiotic fungi *Trichoderma atroviride*, *Trichoderma asperellum*, and necrotrophic pathogenic fungi *Monilinia fructicola*, *Botrytis cinerea*, and *S. sclerotiorum*, while SS1G_13809 is only distributed in necrotrophic pathogenic fungi *Botrytis cinerea*, *S. sclerotiorum*, *Marssonina coronariae*, *Monilinia fructicola*, *Chlorociboria aeruginascens*, etc. (Fig. S6).

**Fig 7:**
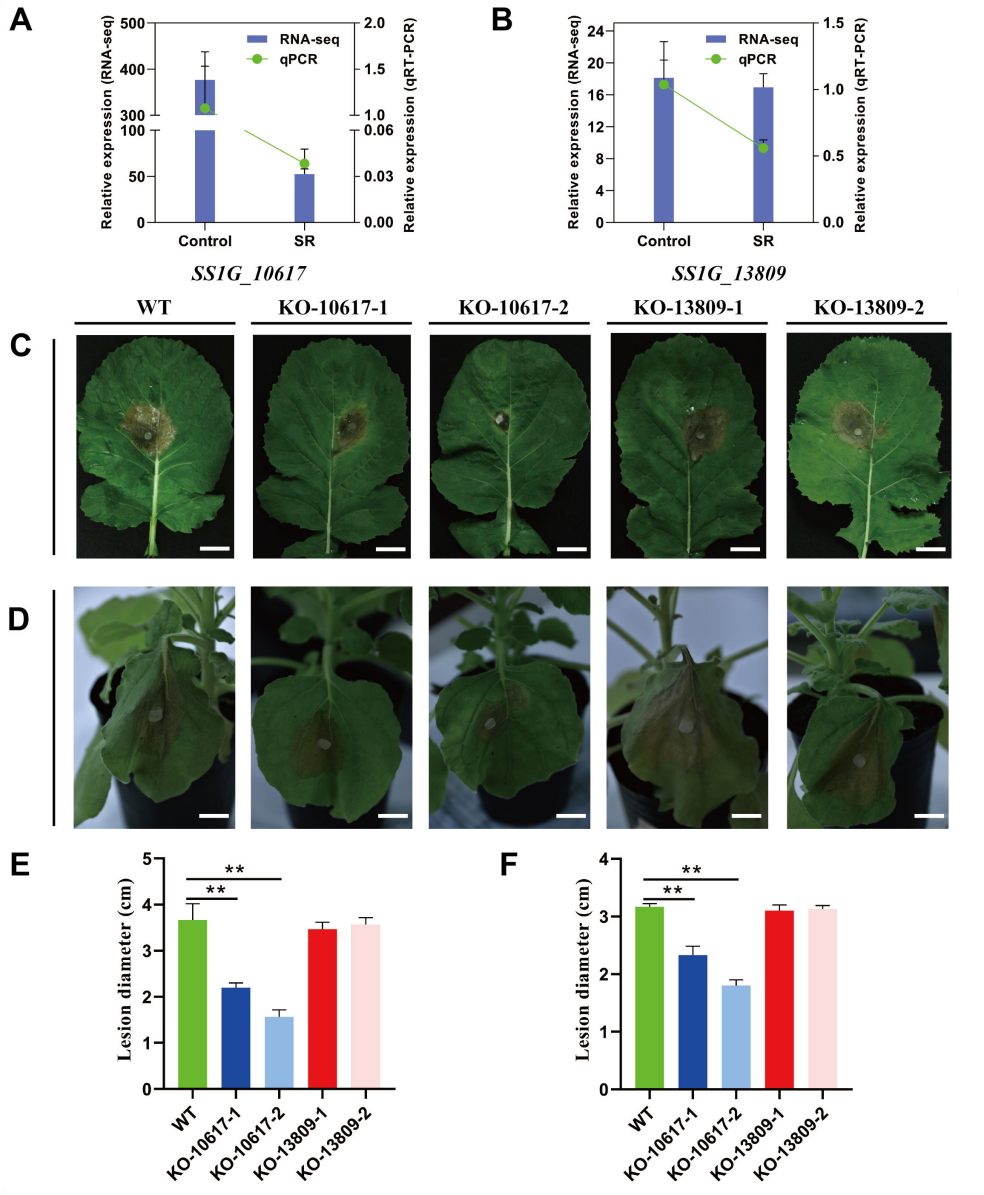
Virulence assay of KO-10617 and KO-13809 on symptomatic host plants (*B. napus* and *N. benthamiana*). Expression levels of *SS1G_10617* (**A**) and *SS1G_13809* (**B**) in rapeseed stalks at 2 dpi with *S. sclerotiorum. S. sclerotiorum* hyphae on half-strength MS agarose medium for 2 days were used as controls. The virulence of the wild-type strain, KO-10617, and KO-13809 were assayed on the detached *B. napus* (**C**) and living *N. benthamiana* (**D**) leaves. Strains were inoculated on rapeseed leaves for 2 days at 20°C. Lesion diameters were measured on the detached *B. napus* (**E**) and living *N. benthamiana* (**F**) leaves at 2 dpi. Error bars represent the SD. **P < 0.01 (*n*  =  5, five independent experiments).

To determine the biological function of *SS1G_10617* and *SS1G_13809* in *S. sclerotiorum*, knockout (KO) mutants of *SS1G_10617* and *SS1G_13809* and complementation strains were obtained and verified by PCR or RT-PCR (Fig. S7). *SS1G_10617* mutants were termed KO-10617-1 and KO-10617-2, and *SS1G_13809* mutants were termed KO-13809-1 and KO-13809-2. To evaluate the role of *SS1G_10617* and *SS1G_13809* in *S. sclerotiorum* development and the ability to degrade starch, colony morphology on the glucose medium, starch medium, and starch medium with glucose was observed. The vegetative growth of the KO-10617 was remarkably inhibited on the glucose medium and that of KO-13809 mutants was not influenced compared to the wild-type strain (Fig. 5C through E). Furthermore, the ability of KO-10617 and KO-13809 to degrade starch was significantly decreased compared to that of the wild-type strain (Fig. 5C and F). Noteworthily, the growth rate of mutants was restored partially when glucose was added to the starch medium (Fig. 5C, G, and H). To further evaluate the role of *SS1G_10617* and *SS1G_13809* during the colonization of *S. sclerotiorum* on wheat plants, the relative biomass of the wild-type strain, KO-10617, and KO-13809 was detected by qPCR in wheat roots at 12 dpi. The qPCR results showed that the ability to colonize wheat roots of KO-10617 and KO-13809 was significantly decreased compared to that of the wildtype (Fig. 6C and D). Again, fluorescence observation also confirmed the different colonization among the strains (Fig. 6E). Interestingly, *SS1G_10617* and *SS1G_13809* were downregulated at 2 dpi during colonization of the rapeseed stalks (Fig. 7A and B). Furthermore, to analyze the role of *SS1G_10617* and *SS1G_13809* in pathogenicity, the individual strains of *S. sclerotiorum* were inoculated on the symptomatic host detached rapeseed and living *Nicotiana benthamiana* leaves. KO-10617 mutant had dramatically reduced pathogenicity compared to that of the wildtype. Surprisingly, the lesion caused by the KO-13809 showed no significant difference (Fig. 7C through F). An SEM analysis was carried out and the results showed that these two mutants formed appressoria during infection in *N. benthamiana* leaves, whereas they did not form the structure on wheat roots (Fig. S8A through D). Complementation of the strains KO-10617 and KO-13809 restored the growth rate, virulence, and colonization capacity to the level of the WT strain (Fig. S9A through C).

Collectively, our results showed that *SS1G_10617* is involved not only in modulating the symbiotic relationship between *S. sclerotiorum* and wheat plants but also in regulating the virulence of symptomatic host plants. *SS1G_13809* is only involved in modulating the symbiotic relationship between *S. sclerotiorum* and wheat plants. These results further suggested that starch may be responsible for the major carbon source of *S. sclerotiorum* in the symbiosis with wheat plants.

## DISCUSSION

Schizotrophism is the destructively pathogenic or mutualistically endophytic tropism of fungi that depends on the host (19). Here, we found a distinct difference in the phenotypes of different hosts during *S. sclerotiorum* colonization. For instance, rapeseed stems show typical necrosis of SSR, whereas wheat roots remain endophytic. Furthermore, appressoria are required for *S. sclerotiorum* colonization in the symptomatic rapeseed host, but not in the endophytic host wheat. These phenotypic and mycelial morphology distinctions may be based on the myriad of host-dependent transcriptional programs. How *S. sclerotiorum* reprograms its development and physiological processes to adapt to and counteract plant defenses and immunity in the symptomatic host rapeseed and endophytic host wheat remains a mystery and is the subject of current studies.

Previously, comparative transcriptomic investigations uncovered that *F. virguliforme* extensively reprograms its transcriptional landscape to adopt a different lifestyle rather than expressing unique transcripts in the symptomatic host soybean or the endophytic host maize (9). In the current study, we observed that the potential conservative pathways, including carbohydrate metabolic process, carbohydrate transport, as well as polysaccharide, organic substance, cellulose, lipid, and phospholipid catabolic process, were commonly induced during *S. sclerotiorum* colonization in both endophytic and symptomatic fungal transcriptomes. It is worth noting that these processes related to carbohydrate metabolism and transport are critical for fungal growth (23, 43). Regardless of the host, phospholipid catabolism is a crucial mechanism for the synthesis of secondary metabolites that enable fungal colonization (28, 29). Furthermore, nutrient acquisition and growth of fungi are also linked to catabolic activity (25). Overall, it is not surprising that these potential conservative pathways are critical for fungal growth and infection, each of which coincided with changes in fungal access to nutrient sources.

Some fungal phytopathogens have evolved appressoria to infect their hosts. Our study demonstrated that appressoria are required for *S. sclerotiorum* colonization in the symptomatic host rapeseed, but not in the endophytic host wheat. Autophagy, cell cycle process, and cytochrome complex assembly in fungal hyphae support the association with the formation of infection structures like appressoria (31 – 33). NAD metabolic process has been previously associated with hyphal differentiation initiation for infection structures (34, 35). Noteworthily, autophagy, cell cycle process, cytochrome complex assembly, and NAD metabolic process were enriched in symptomatic host rapeseed, yet not in endophytic host wheat. This is consistent with what we observed in the development of appressoria-like structures during the early infection stages of *S. sclerotiorum* in rapeseed plants. Hence, *S. sclerotiorum* might invade the endophytic host wheat root possibly via natural openings between epidermal cells or penetrate directly into the root epidermal cells through hyphae rather than appressoria.

OA is a common low-molecular-weight organic acid produced by living organisms and is essential for fungal pathogenicity, nutrient uptake, and metal tolerance (49). Moreover, endophytic fungi colonization could alter the concentration of OA in host plants, such as OA concentration was significantly elevated in the mycorrhizal symbiosis maize plant leaves than in the nonmycorrhizal maize plants (50). During the early infection phases of *S. sclerotiorum* in symptomatic host plants, OA inhibits the oxidative burst, callose deposition, and host defenses, and induces the production of host reactive oxygen species, which in turn causes host cell death (25). Interestingly, our results suggest that *Ss-oah1* and *Ss-pth2*, which positively control oxalate production, were upregulated during rapeseed stem colonization, conversely, on wheat, these two genes as well as positive transcription factor gene *Ss-pac1* were downregulated. During rapeseed stem colonization, the oxalate decarboxylase enzyme gene *Ssodc2* was downregulated, whereas, it was significantly upregulated on wheat. This finding implies that *S. sclerotiorum* may alter the concentration of OA and increase the ambient pH in wheat plant cells by upregulating *Ss-oah1* and *Ss-pth2*, as well as *Ss-pac1*, and downregulating *Ssodc2* during colonization on endophytic host wheat. Hence, OA is a critical element for the colonization of *S. sclerotiorum* in the symptomatic host rapeseed, while not in the endophytic host wheat. Additionally, it is further speculated that OA may be one of the critical factors regulating the phenotype of hosts and the transformation of *S. sclerotiorum* tropism adapting to symptomatic and endophytic hosts.

Both pathogenic and mutualistic fungi deploy effector proteins during plant infection to manipulate the host immune response. Some effectors are extremely specific for a unique pathogen species, while others are conserved across a wide variety of pathogens and endophytes (51, 52). For instance, two early root colonization (ERC) effectors of *Fusarium oxysporum*, ERC1 and ERC3, can reduce virulence and activate host plant immune responses, while ERC mutant in a nonpathogenic strain reduced root colonization and biocontrol ability (53). Small molecule biosynthesis is also potentially associated with the function of necrotrophic effectors (37, 38). In this study, several *S. sclerotiorum* SP encoding genes were uniquely upregulated during rapeseed stem colonization, among which *SsCP1*, *Ssv263*, *Ss-Bi1*, *Ss-cmu1,* and *SsSSVP1* have been demonstrated to induce cell death and promote disease in the symptomatic host (16, 40 – 43). These results are also consistent with the fact that *S. sclerotiorum* had already begun a necrotrophic infection on rapeseed stems. Strikingly, 67 SP encoding genes, including *SsSSVP1*, were also induced during wheat root colonization, besides in rapeseed stem. We hypothesize that these SPs might also modulate the invasion of *S. sclerotiorum* and manipulate the plant immune system on both symptomatic host rapeseed and endophytic host wheat. Additionally, 68 and 87 uniquely upregulated SP encoding genes from *S. sclerotiorum* were detected during rapeseed stem and wheat root colonization, which may be critical elements for the schizotrophic mechanism of *S. sclerotiorum*. However, the function of these uniquely upregulated SP encoding genes in manipulating the plant immune system on both symptomatic host rapeseed and endophytic host wheat still needs to be uncovered.

Starch is composed of two kinds of polysaccharides with different structures, amylose, and amylopectin, and is also the main form of carbohydrate storage in plants (54). Many starch-degrading enzymes have already been isolated from archaea, bacteria, and fungi. α-amylase, β-amylase, and glucoamylase are known to break α-1,4-linkages of starch to produce several types of oligosaccharides, maltose, and glucose. Pathogenic and endophytic fungi use starch as the carbon source to provide nutrition for their growth and development during colonization (55, 56). In this investigation, we discovered that many genes related to carbohydrate metabolism and starch and sucrose metabolism were uniquely upregulated during colonization of the endophytic host wheat root, particularly, *SS1G_10617* and *SS1G_13809*, in the degradation of starch to glucose pathway. Furthermore, *SS1G_10617* and *SS1G_13809* mutants displayed abnormal starch degradation and showed a lower ability to colonize the endophytic host wheat root. Interestingly, *SS1G_10617* was proved to be involved in modulating the pathogenicity of *S. sclerotiorum* on symptomatic hosts. *SS1G_10617* mutant had a severe abnormal starch degradation and colonization ability than did *SS1G_13809* mutant, suggesting that *SS1G_10617* could play a central role during the colonization of *S. sclerotiorum* in the endophytic host wheat root. It is proposed that *SS1G_10617* and *SS1G_13809*, components in the starch degradation pathway, are involved in modulating colonization ability in *S. sclerotiorum* by mediating starch degradation. Furthermore, it prompted us to propose that starch may be responsible for the major carbon source of *S. sclerotiorum* in the symbiosis between *S. sclerotiorum* and wheat plants.

In the previous study, we speculated that schizotrophic *S. sclerotiorum* has two distinct niches within agroecosystems and a potential role in regulating the species compositions of natural ecosystems (19). Based on these observations, we surmise that the comparative analysis of the interaction of *S. sclerotiorum* with two hosts supports our hypothesis of a divergence in the transcriptome of *S. sclerotiorum*. In addition, this study demonstrates that these gene expression profiles highlight the transcriptional divergence plasticity of a single fungal isolate on multiple hosts. In this regard, the analysis highlights the significance of rewiring during host-*S. sclerotiorum* interactions, including the expression of distinct gene networks underpinning the development of endophytic and symptomatic programs. Furthermore, we speculate that schizotrophic endophyte *S. sclerotiorum* in the endophytic host will modulate the unique expression pattern of genes to attack the symptomatic host. Conversely, schizotrophic pathogen *S. sclerotiorum* in the symptomatic host will modulate the unique expression pattern of genes to adapt endophytic growth in the endophytic host when the symptomatic host is lacking or absent. As a further link to the work described herein, the function of this unique expression pattern of genes will need to be verified.

In summary, we observed a general increase in the gene expression associated with pathogenicity in rapeseed, including small protein secretion, appressorial formation, and OA toxin production. Conversely, in wheat, many carbohydrate metabolism and transport-associated genes were induced, indicating a general increase in processes associated with carbohydrate acquisition. Furthermore, we confirmed that appressorium is required for *S. sclerotiorum* colonization symptomatic hosts rather than endophytic wheat. In addition, *SS1G_10617* and *SS1G_13809*, in the degradation of starch to glucose pathway, showed a lower ability to colonize on the endophytic host wheat root. Interestingly, *SS1G_10617* is also associated with pathogenicity in symptomatic hosts. Hence, this may be a possible reason why there is not a shift from biotrophy to necrotrophy in wheat. This study contributes to a better understanding of schizotrophic fungi and provides new clues for cultivating disease-resistant varieties and adjusting reasonable farming strategies.

## MATERIALS AND METHODS

### Plant and fungal materials, maintenance, and preparation

The wheat (*Triticum aestivum*) cultivar Zheng 9023 and the rapeseed (*Brassica napus*) cultivar QingYou 1 were purchased from the commercial seed market in Chongqing, China. The wild-type strain Ep-1PNA367 of *S. sclerotiorum* was cultured on potato dextrose agar (PDA) plates at 20°C. All transformants were maintained on PDA supplemented with 200 µg/mL hygromycin B (Roche, Switzerland).

Wheat and rapeseed seeds were surface sterilized with 70% ethanol for 2 minutes, 0.5% sodium hypochlorite for 30 minutes, and washed with sterile distilled water three times. The sterilized seeds were sown on half-strength MS agarose medium for 15 days. The wheat and rapeseed seedlings were grown at 20°C (16-h-light/8-h-dark cycle, 70% humidity) in a greenhouse and prepared for RNA-seq and quantification of colonization of *S. sclerotiorum* in wheat roots.

### Sample collection and RNA sequencing

To investigate the differential infection mechanism of *S. sclerotiorum* interacting with the symptomatic host (rapeseed) and endophytic host (wheat), wheat and rapeseed seedlings were grown on half-strength MS agarose medium for 15 days at 20°C and then the wheat roots and rapeseed stalks were collected at 48 hours post-inoculation of WT strain Ep-1PNA367. For the WT strain treatment of the wheat and rapeseed group, five stalks of rapeseed plants and five roots of wheat plants were collected as a sample. The mycelia of the WT strain growing on MS medium for 2 days were used as an *S. sclerotiorum* control group. The samples were immediately placed in liquid nitrogen and ground into powder. To ensure repeatability and remove batch effects, we repeated three independent times under the same growth condition and further randomly mixed the samples from three independent replicates of the same treatment as a sample.

According to the manufacturer’s instructions, total RNA samples were extracted with a TRIzol Plus RNA Purification Kit (Takara, Dalian, China) and treated with RNase-free DNase I (Takara, Dalian, China). The RNA quality was checked using a Nanodrop Spectrophotometer (Thermo Fisher Scientific Inc., Wilmington, DE, USA). RNA integrity was confirmed by 1.5% agarose gel electrophoresis. Two micrograms of total RNA was used for stranded RNA sequencing library preparation using the KC-Digital stranded mRNA library prep kit for Illumina (Catalog no. DR08502; Wuhan Seqhealth Technology Co., Ltd., China) following the manufacturer’s instructions. The kit eliminated the duplication bias during PCR and sequencing steps by using a UMI of eight random bases to label the preamplified cDNA molecules. The library products corresponding to 200–500 bp were enriched, quantified, and finally sequenced on a Novaseq 6000 sequencer (Illumina).

### RNA-Seq data analysis

The adapters, low-quality sequences, and reads with a high concentration of unknown base (N) reads were eliminated to get clean reads. In order to remove the duplication bias introduced during library preparation and sequencing, clean reads were further processed with KC-UID (the official analysis software of Seqhealth Technology Co., Ltd., used to process reads of the UMI RNA-seq library; https://github.com/KC-UID/KC-UID). Clean reads were originally grouped into clusters based on their UMI sequences, where reads sharing the same UMI sequences were assigned in the same cluster. Pairwise alignment was used to compare reads in the same cluster to each other and reads with a sequence identity of over 95% were then removed to a new subcluster. After all the subclusters were generated, multiple-sequence alignments were carried out following the generation of all the subclusters to obtain a consensus sequence for each subcluster. After these procedures, all errors and biases brought about by PCR amplification or sequencing were removed.

The standard RNA-seq analysis employed the deduplicated consensus sequences. The Spliced Transcripts Alignment to a Reference software (default parameters) was used to map them to the reference genome of *S. sclerotiorum* strain 1980 UF-70 (NCBI Genome assembly accession no. ASM14694v2) (57, 58). Gene expression was calculated by the number of reads mapped to the reference genomes using the fragments per kilobase of transcript per million mapped reads method (59). DEGs were selected with FDR < 0.05 and |log_2_ FC| > 1 between the host-induced and control groups. To avoid the noise signals from the high-throughput sequencing, genes only detected in three biological replicates of one condition and above the detection threshold of one count per million were used in this analysis. Based on the annotation of BLAST search results (*E* value < 10^−5^) against three public databases, including the Pfam (http://pfam.xfam.org/), KEGG (http://www.genome.jp/kegg/), and InterPro (http://www.ebi.ac.uk/interpro/) databases, BLAST2GO was used to examine the functional annotation of GO terms. GO enrichment analyses of all genes were performed to examine the biological significance of the genes and GO enrichment analysis with Fisher’s exact tests was performed on differentially expressed transcripts, using *P*-values < 0.01. Using the cluster Profiler package, KEGG enrichment was carried out with the threshold set as *P*-values < 0.05.

### RT-qPCR analysis

The qRT-PCR analysis for validating the different expression data was prepared independently under the same conditions. The cDNA Synthesis SuperMix (TransGen Biotech, China) was used for cDNA synthesis. Gene expression abundance of the target gene was quantified by the qTOWER3G from Analytik Jena Company using the TransStart Green qPCR SuperMix (TransGen Biotech, China). The ubiquitin gene of *S. sclerotiorum* (*SS1G_11035*) and the *TaEF-1a* (Q03033) gene of *Triticum_aestivum* served as internal reference genes. This experiment was repeated with RNA from three biological replicates, with each treatment set having four replicates. Primers for the target genes and internal reference genes were designed using Beacon Designer 8 and are listed in Table S5.

### Multiple alignment, conserved domain identification, and phylogenetical analysis

The sequences of *SS1G_10617* and *SS1G_13809* were retrieved from the NCBI GenBank database (http://www.ncbi.nlm.nih.gov/). The amino acid sequences of SS1G_10617 and SS1G_13809 from *S. sclerotiorum* were used as query sequences to conduct BLASTP. The SignalP-5.0 server (https://services.healthtech.dtu.dk/service.php?SignalP-5.0/) was used to predict signal peptides in SS1G_10617 and SS1G_13809. The conserved functional domains of SS1G_10617 and SS1G_13809 were predicted using the NCBI Conserved Domain Search Tools (https://www.ncbi.nlm.nih.gov/Structure/cdd/wrpsb.cgi) and SMART (http://smart.embl-heidelberg.de/).

For phylogenetic analysis, alignments were performed with SS1G_10617 and SS1G_13809 in *S. sclerotiorum* and their homologs using the MUSCLE program of MEGA 7 (https://www.megasoftware.net/) with default parameters. Thereafter, the phylogenetic tree was constructed in MEGA 7 using the maximum-likelihood method with a bootstrap value of 1,000 replicates.

### Gene deletion

The strategy based on a split-marker system was used to obtain *SS1G_10617* and *SS1G_13809* gene knockout mutants (40). Two approximately 0.8-kb fragments gene-5′ and gene-3′ were amplified from *S. sclerotiorum* genomic DNA using PrimeSTAR HS DNA Polymerase (Takara, Shiga, Japan) with primer pair gene-*Sma*I-Down-F/ gene-*Kpn*I-Down-R, and then cloned into vector pSKH using the Hieff Clone Plus One Step Cloning Kit (Yeasen, Shanghai, China).

The 2.1-kb hygromycin phosphotransferase (*hph*) gene from vector pSKH was used as a template to clone the front sequences of hph, which was termed HP, and the rear sequences of hph, termed PH. The upstream flanking sequence of the gene was fused with HP using PCR with primers gene-*Not I*-Up-F/hp-R, and the downstream flanking sequence of the gene was fused with PH using primers ph-F/gene-*Kpn I*-Down-R. The two overlapping fragments were concurrently transformed into the protoplasts of the *S. sclerotiorum* wild-type strain. Hygromycin-resistant transformants were selected in a regeneration agar medium with 150 µg/mL hygromycin B and screened using PCR. Primer pairs gene-Up-F/Up-R/Down-F/gene-Down-R were used to screen knockout transformants. Primer sequences for plasmid construction are listed in Table S6.

### Construction of the KO-10617 and KO-13809 complementation strains

For complementation of the KO-10617 and KO-13809 mutant, the 4.15-kb PCR product containing a 0.98-kb upstream sequence (which contained the endogenous promoter), the full-length *SS1G_10617* or *SS1G_13809* gene coding region, and a 0.80-kb downstream sequence was amplified from strain Ep-1PNA367 (WT) genomic DNA using primers C10617F and C10617R (or C13809F and C13809R) and cloned into the *Xho*1 and *Sac*1 sites of pCETNS to generate the complementary vector pCETNS-10617. The fragment was concurrently transformed into the protoplasts of the *S. sclerotiorum* KO-10617 strain. G418 and hygromycin-resistant transformants were selected in a regeneration agar medium with 150 µg/mL hygromycin B and 100 µg/mL G418 and screened using PCR. Primer pairs Co-F and Co-R were used to screen knockout transformants. Primer sequences for plasmid construction are listed in Table S6.

### Microscopic observation

To observe the growth of *S. sclerotiorum* on the wheat and rapeseed by confocal microscopy, the rapeseed stalk was inoculated with 1/2 mycelial agar discs (Ф = 3.0 mm) of the WT strain. All *S. sclerotiorum* transformants were shake-flask cultured in potato dextrose broth medium (PDB) for 3 days at 20°C, 200 rpm; then, the *S. sclerotiorum* hyphal fragment suspension from the homogenizer was diluted to OD_600_ = 2.4 with 1/10 plant nutrition medium (PNM) [KNO_3_, 5.06 g/L; KH_2_PO_4_, 5.00 g/L; K_2_HPO_4_, 2.51 g/L; MgSO_4_, 24.07 g/L; Ca(NO_3_)_2_, 4.72 g/L; FeSO_4_·7H_2_O, 0.14 g/L; NaCl, 2.50 g/L; Na_2_EDTA 2H_2_O, 0.20 g/L; MES 1.00 M, at pH 6.0]. Wheat seedlings and the hyphal fragment suspensions were then used to inoculate the prepared 100 mL 1/10 PNM (2.5 mL of hyphal fragment suspension/100 mL 1/10 PNM) mixed with 0.05% (vol/vol) Tween 20 in a sterile jar. Wheat seedlings were grown at 20°C (16-h-light/8-h-dark cycle, 70% humidity) in a greenhouse. Wheat roots colonized with *S. sclerotiorum* 12 dpi were washed carefully with water five times and incubated in a 10 µg/mL wheat germ agglutinin (WGA) conjugated to fluorescein 5-isothiocyanate (FITC) (Sigma) and 1 µg/mL propidium iodide (PI) in the dark for 1 hour at room temperature according to the manufacturer’s instructions before imaging by a LEICA confocal microscope (LEICA SP8). FITC and PI fluorescence were visualized at 488 and 561 nm excitation and emission were detected at 495–540 and 570–640 nm, respectively.

For further observation with TEM, 5-mm root segments from wheat seedlings grown on MS medium for 12 days after inoculation with *S. sclerotiorum* WT strain and Δ*Ss-caf1* were fixed in 0.4% (vol/vol) glutaraldehyde solution overnight at 4°C. After washing in phosphate-buffered saline (PBS) buffer, roots were dehydrated with a graded ethanol series. Samples were then embedded in Epon-821 and polymerized at 60°C. Thin sections (50 nm) were cut using a Leica ULTRACUT UCT ultramicrotome with a diamond knife.

### Starch degradation assay

To evaluate fungal growth characteristics and the ability of starch degradation, fresh hyphal plugs (5 mm in diameter) of all strains were inoculated on glucose media (glucose, 40 or 80 g/L; yeast extract, 0.4 g/L; and agar, 11 g/L), and starch media (soluble starch, 20 g/L; yeast extract, 0.4 g/L; MgSO_4_·7H_2_O, 0.5 g/L; KCl, 0.5 g/L; NaNO_3_, 2.0 g/L; FeSO_4_·7H_2_O, 0.01 g/L; KH_2_PO_4_·3H_2_O, 0.5 g/L; agar, 11 g/L, at pH 5.0) and starch media with glucose (soluble starch, 20 g/L; glucose, 40 or 80 g/L; yeast extract, 0.4 g/L; MgSO_4_·7H_2_O, 0.5 g/L; KCl, 0.5 g/L; NaNO_3_, 2.0 g/L; FeSO_4_·7H_2_O, 0.01 g/L; KH_2_PO_4_·3H_2_O, 0.5 g/L; agar, 11 g/L, at pH 5.0) at 20°C for 5 days. The colony diameters were measured every 12 hours. The experiment was repeated three times, with a set of four replicates.

### Pathogenicity assays

*B. napus* (QingYou 1) and *Nicotiana benthamiana* were used for the pathogenicity assay of *S. sclerotiorum* strains. Fresh hyphal plugs (5 mm in diameter) were inoculated on the detached leaves of rapeseed or living *N. benthamiana* plants. The inoculated detached leaves or living plants were maintained at 90% relative humidity in a greenhouse. The wild-type strain was used as a control under the same conditions. The diameters of lesions on rapeseed leaves were recorded at 2 dpi. The experiment was repeated three times with five replicates.

### Quantification of *S. sclerotiorum* colonization by qPCR

For quantifying the root colonization, wheat seedlings were collected from sterilized seeds on half-strength MS agarose medium for 15 days. *S. sclerotiorum* wild-type strain, KO-10617, and KO-13809 were shake-flask cultured in PDB for 3 days at 20°C, 200 rpm; then, the *S. sclerotiorum* hyphal fragment suspension from the homogenizer was diluted to OD_600_ = 2.4 with 1/10 PNM. The hyphal fragment suspensions were then used to inoculate the prepared 100 mL 1/10 PNM (2.5 mL of hyphal fragment suspension/100 mL 1/10 PNM) mixed with 0.3% (vol/vol) Tween 20 in a sterile jar (five wheat seedlings/jar). For each set of experiments, four biological replicates with five seedlings per replicate were used and cultivated at 20°C (16-h-light/8-h-dark cycle, 70% humidity) in a greenhouse. As a mock, 1/10 PNM inoculated roots were used. Wheat seedling roots colonized with *S. sclerotiorum* (12 dpi) and control wheat seedlings were thoroughly washed with tap water 10 times and sterilized with 70% ethanol for 1.5 minutes, 2.5% sodium hypochlorite for 30 minutes, and washed with sterile distilled water three times to remove fungal hyphae from the root surface.

Wheat seedling roots were collected and used for DNA extraction. Relative *S. sclerotiorum* biomass in wheat root was quantified by the qTOWER3G from Analytik Jena Company using the TransStart Green qPCR SuperMix (TransGen Biotech, China). PCR amplification was performed under the following conditions: 95°C for 3 minutes, followed by 43 cycles of 95°C for 15 s, 57°C for 15 s, and 72°C for 20 s. Melt curve profiles were analyzed for each gene tested at the end of each PCR reaction. The ubiquitin gene of *S. sclerotiorum* (*SS1G_11035*) and the *TaEF-1a* (Q03033) gene of *T._aestivum* served as internal reference genes. This experiment was repeated at least three times, with each treatment set having four replicates. Primers for the target genes and internal reference genes were designed using Beacon Designer 8 and are listed in Table S6.

### Statistical analyses

The data of fungal growth rate and pathogenicity assay, as well as quantification of colonization ability were subjected to one-way ANOVA or two-way ANOVA. These data were used for variance analysis in SPSS (SPSS 19), and the error bars representing the standard deviations (SD) in this study.

## Data Availability

All sequence reads generated in this study were deposited in the NCBI GEO database with accession numbers: GSM6759258, GSM6759259, GSM6759260, GSM6759261, GSM6759262, GSM6759263, GSM6759264, GSM6759265, and GSM6759266.

## SUPPLEMENTAL MATERIAL

Supplemental figures spectrum.02612-23-s0001.pdf Fig. S1 to S9.

Supplemental tables spectrum.02612-23-s0002.xlsx Tables S1 to S6.

ASM does not own the copyrights to Supplemental Material that may be linked to, or accessed through, an article. The authors have granted ASM a non-exclusive, world-wide license to publish the Supplemental Material files. Please contact the corresponding author directly for reuse.
