# Supplementary material for: Transcriptional plasticity of schizotrophic Sclerotinia sclerotiorum responds to symptomatic rapeseed and endophytic wheat hosts
Source: Microbiol Spectr. 2023 Oct 31;11(6):e02612-23. doi: 10.1128/spectrum.02612-23 (PMC10714719; doi:10.1128/spectrum.02612-23)
Supplement: Supplemental figures — Fig. S1 to S9. [file spectrum.02612-23-s0001.pdf]

Supplementary Figures

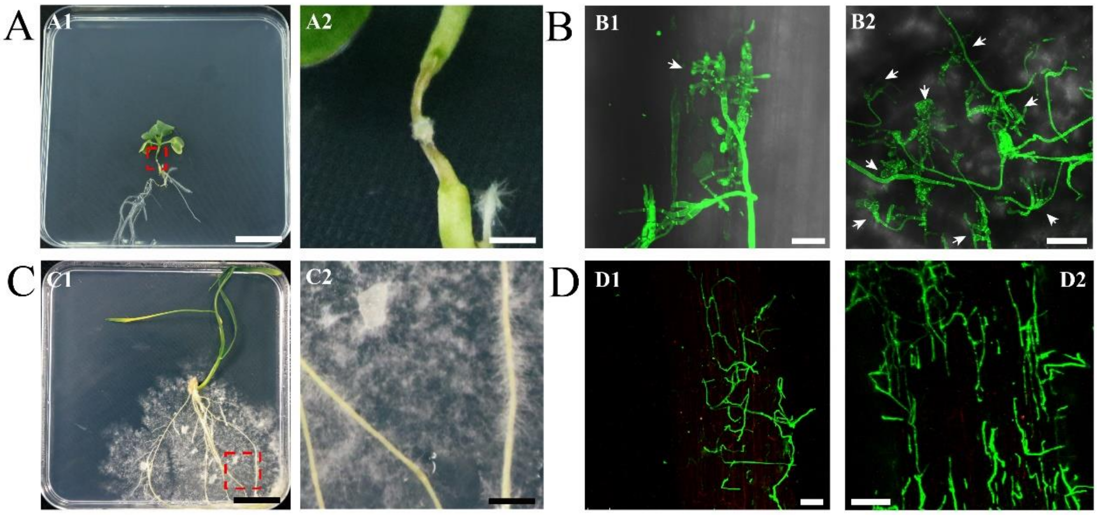

Fig. S1 The phenotypes and mycelial morphology of *Sclerotinia sclerotiorum* at the early stages of colonization of symptomatic rapeseed host and asymptomatic wheat host. (A) Representative images of rapeseed plant stem inoculated with *S. sclerotiorum* WT strain at 2 dpi; (A2) The enlargement of the boxed region in (A1); Scale bars for A1 and A2 are 2 cm and 25 mm. (B) Visualized mycelial morphology of *S. sclerotiorum* at the early stages of colonization of rapeseed stems by confocal microscopy; (B1) and (B2) Photographs were taken at 24 hours post inoculation (hpi) and 36 hpi, respectively. Hyphae of WT strain were stained with WGA. White arrows indicate appressoria of *S. sclerotiorum*; Scale bars for B1 and B2 are 50 μm. (C) Representative images of wheat root inoculated with *S. sclerotiorum* WT strain at 2 dpi; (C2) The enlargement of the boxed region in (C1); Scale bars for C1 and C2 are 2 cm and 30 mm. (D) Visualized mycelial morphology at the early stages of colonization of wheat roots inoculated with the *S. sclerotiorum*-WT strain by confocal microscopy; (D1) and (D2) Photographs were taken at 24 hpi and 48 hpi, respectively. Scale bars for D1 and D2 are 80 μm and 200 μm. Hyphae of WT strain were stained with WGA and wheat plant cell-wall apoplastic space was stained with PI.

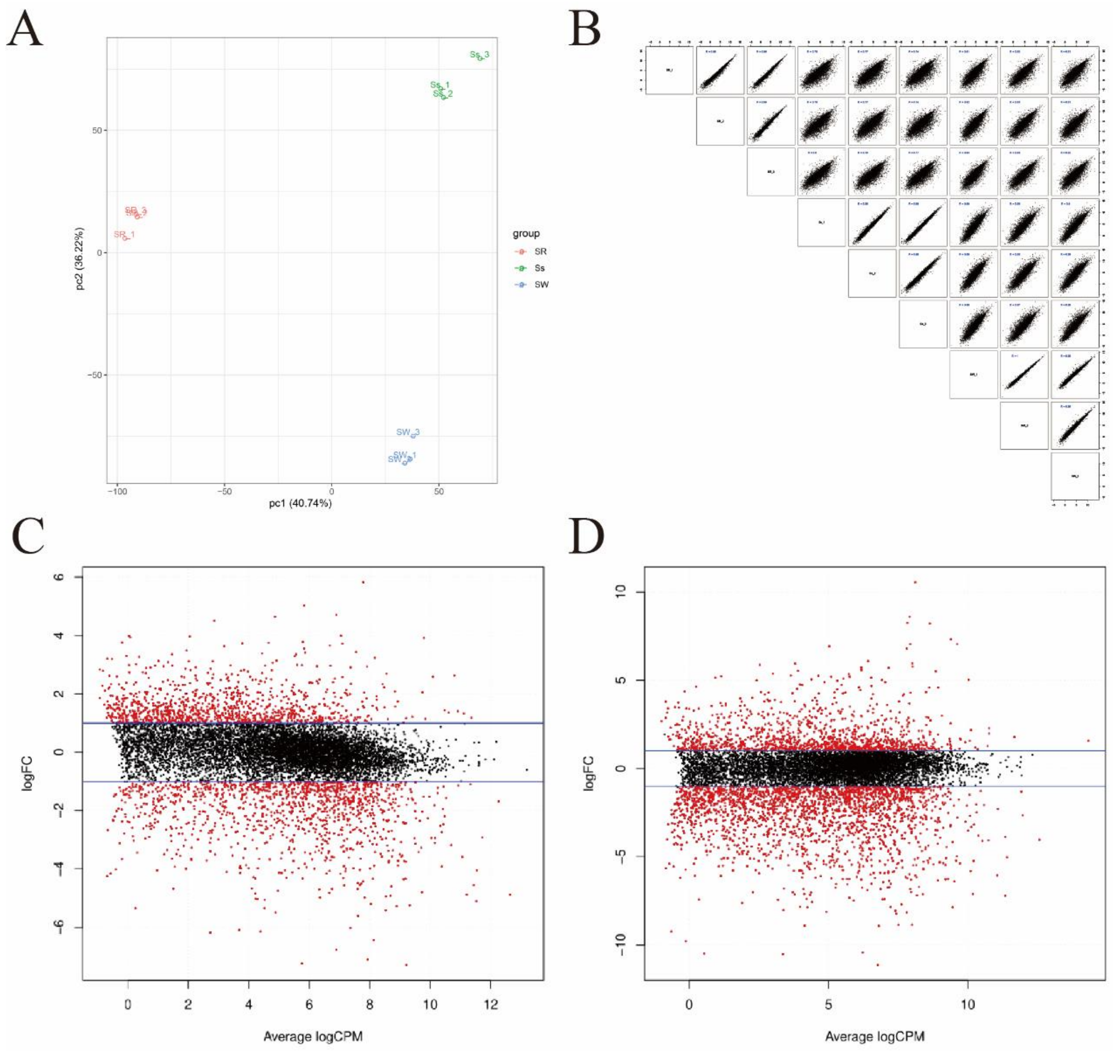

Fig. S2 Transcriptome profile of digital RNA-Seq data.

(A) Principal-component analysis (PCA) of unique molecular identifier–RNA sequencing data. (B) Sample RPKM correlation analysis for each experiment. (C) and (D) MA-plot of the differential expression analysis for SR or SW with SS, respectively.

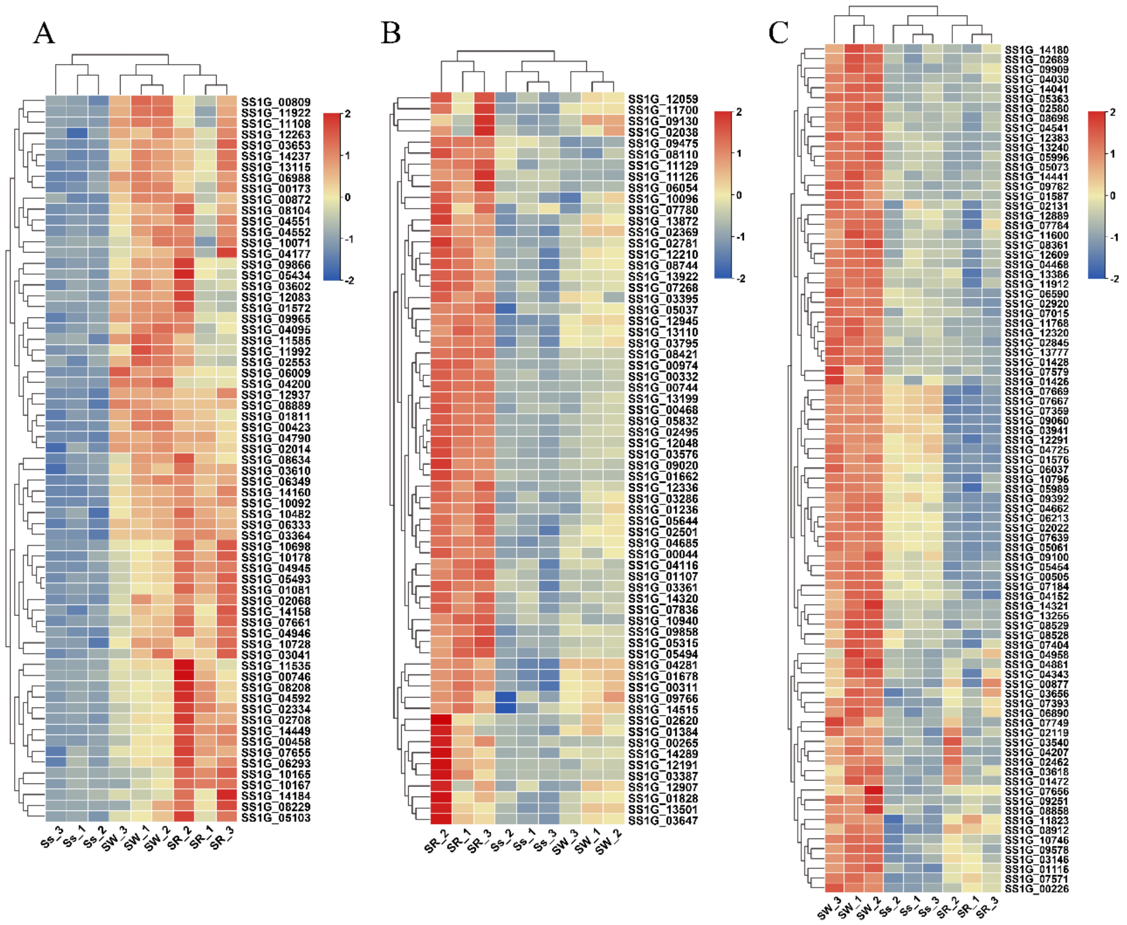

Fig. S3 Expression profiles of *Sclerotinia sclerotiorum* SP encoding genes during colonization of rapeseed and wheat. (A) Heat maps of significant commonly up-regulated *S. sclerotiorum* SP encoding genes ($|\log_2 FC| > 1$, FDR < 0.05) during the colonization of rapeseed and wheat plant (n =67) (B and C) Heat maps of significant uniquely up-regulated *S. sclerotiorum* SP encoding genes ($|\log_2 FC| > 1$, FDR < 0.05) during the colonization of rapeseed (B) (n = 66), and wheat root (C) (n = 87).

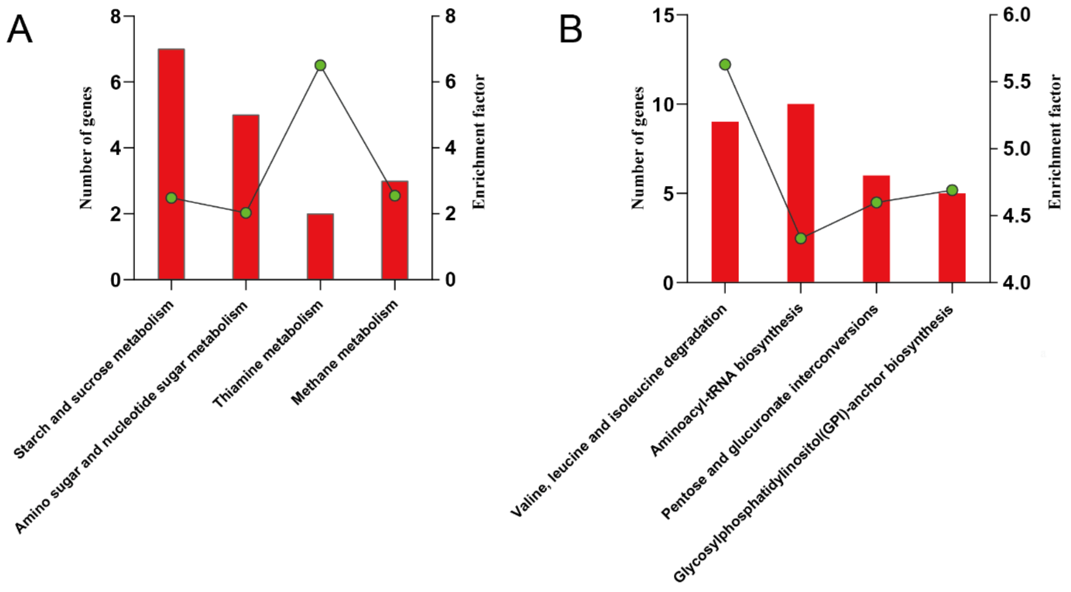

Fig. S4 Kyoto Encyclopedia of Genes and Genomes (KEGG) enrichment analyses of uniquely upregulated and downregulated *Sclerotinia sclerotiorum* genes when colonization of wheat and rapeseed. (A) Enriched KEGG pathways of uniquely upregulated genes during *S. sclerotiorum* colonizing wheat. (B) Enriched KEGG pathways of uniquely upregulated genes during *S. sclerotiorum* colonizing rapeseed.

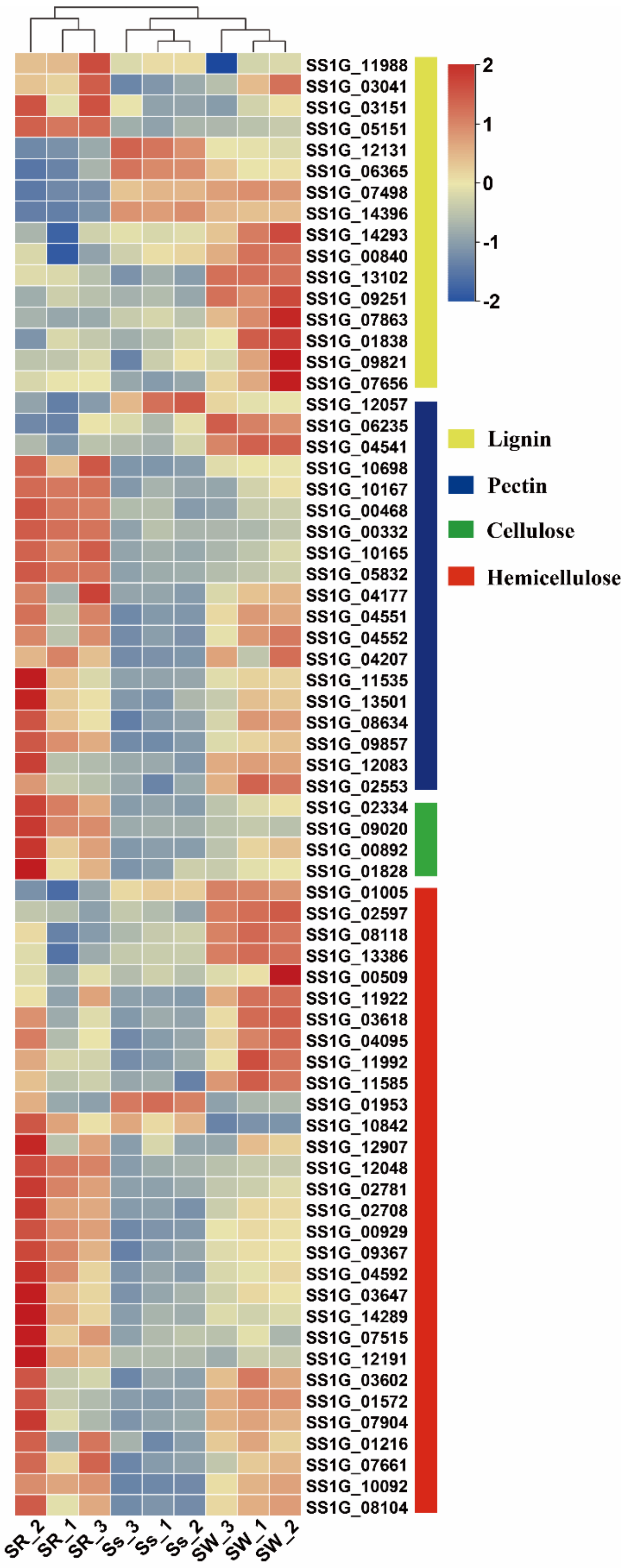

Fig. S5 Expression profiles of *Sclerotinia sclerotiorum* PCWEs during the colonization of rapeseed and wheat.

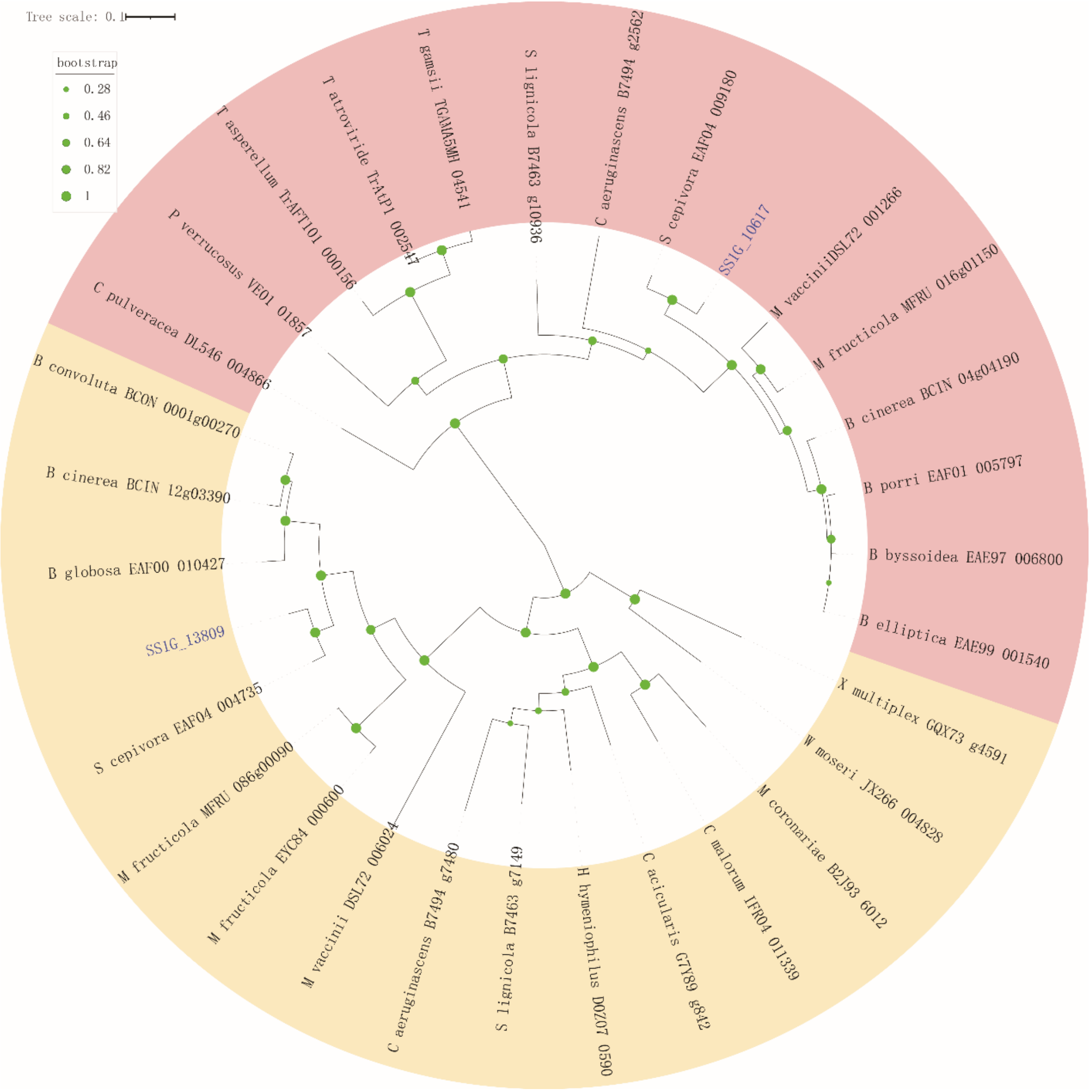

Fig. S6 Phylogenetic analysis of SS1G_10617 and SS1G_13809 proteins in typical species.

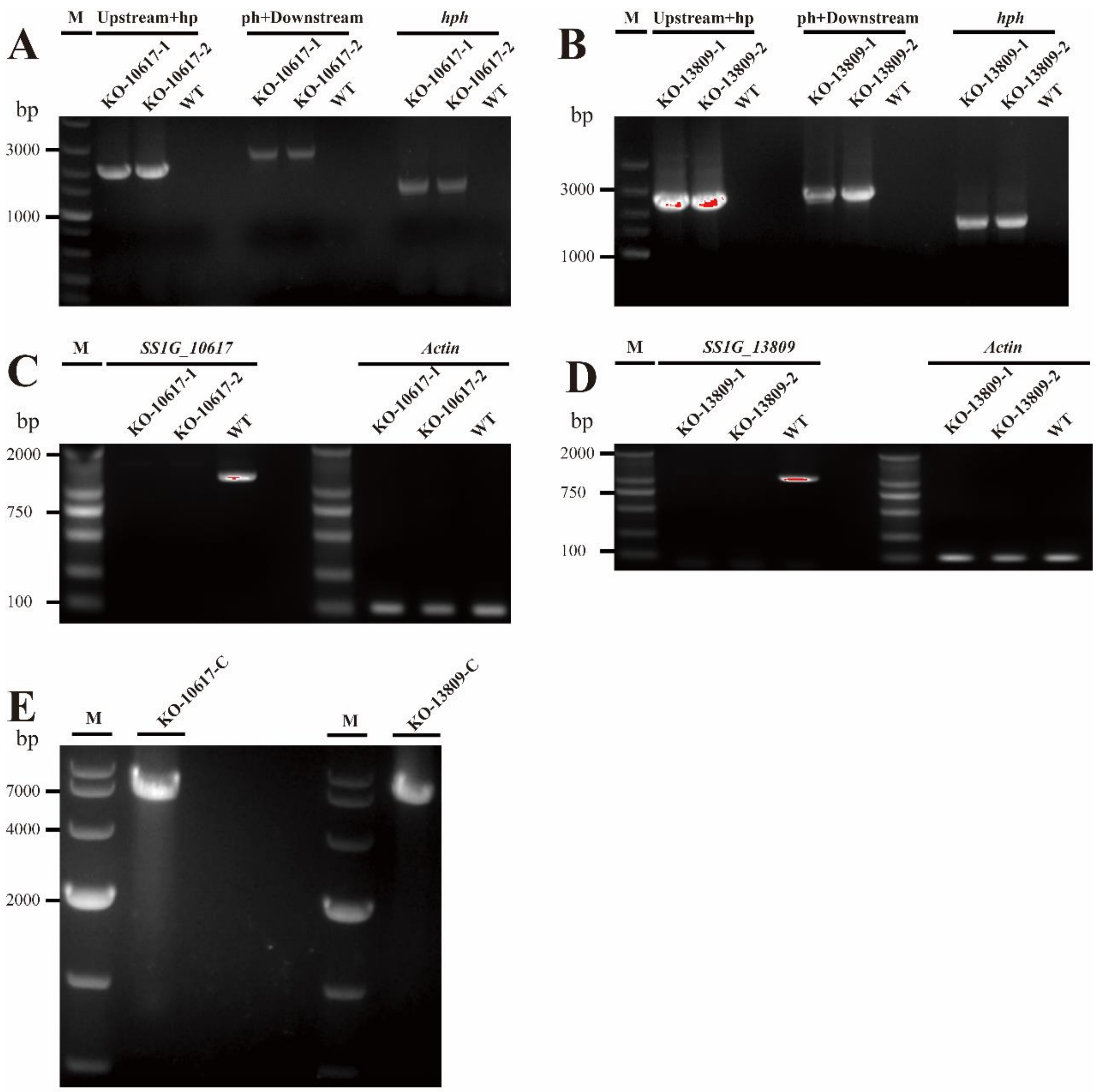

Fig. S7 Verification of KO-10617 and KO-13809 mutants, and complementation strains.

(A and B) PCR verification of KO-10617 mutants (A) and KO-13809 mutants (B). (C and D) RT-PCR verification of the KO-10617 mutants (C) and KO-13809 mutants (D). The ubiquitin gene of *S. sclerotiorum* (*SS1G_11035*) served as an internal reference gene. (E) PCR verification of KO-10617 and KO-13809 complementation strains.

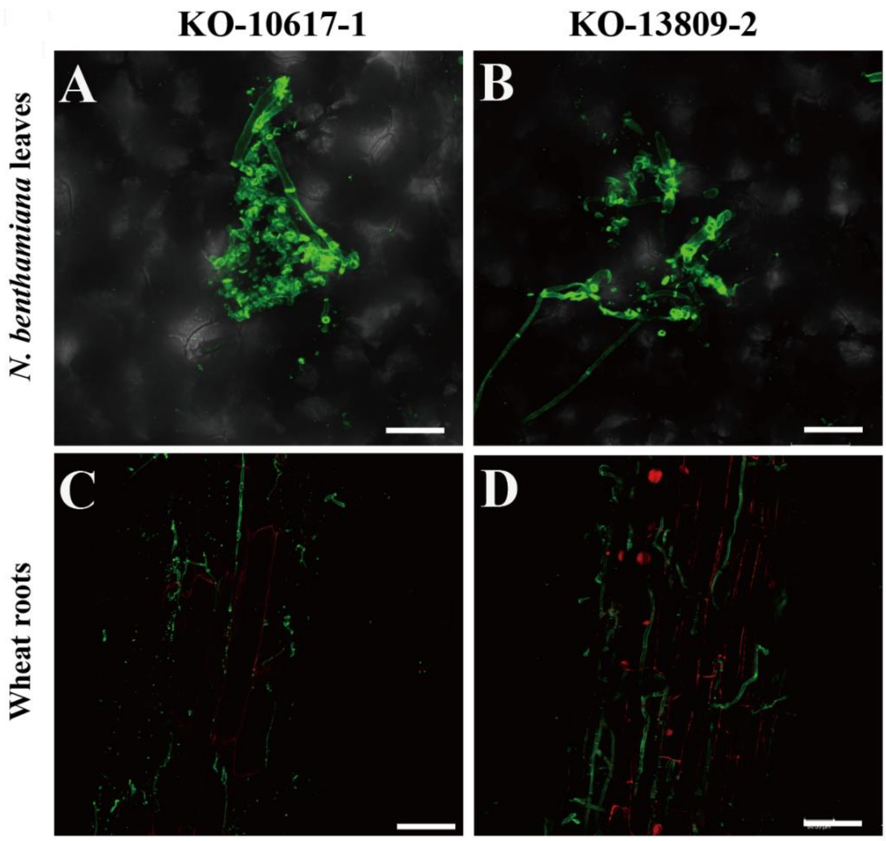

Fig. S8 The mycelial morphology of the KO-10617 and KO-13809 mutants colonizing symptomatic *N. benthamiana* leaves and asymptomatic wheat roots.

(A and B) Representative images of *N. benthamiana* leaves inoculated with the KO-10617 and KO-13809 mutants at 10 hpi, respectively; Hyphae of KO-10617 and KO-13809 mutant strains were stained with WGA. Scale bars for A and B are 25 μm and 20 μm. (C and D) Visualized mycelial morphology of the KO-10617 and KO-13809 mutants at 18 dpi of colonization of wheat roots by confocal microscopy, respectively. Hyphae of KO-10617 and KO-13809 mutant strains were stained with WGA and wheat plant cell-wall apoplastic space was stained with PI. Scale bars for C and D are 100 μm.

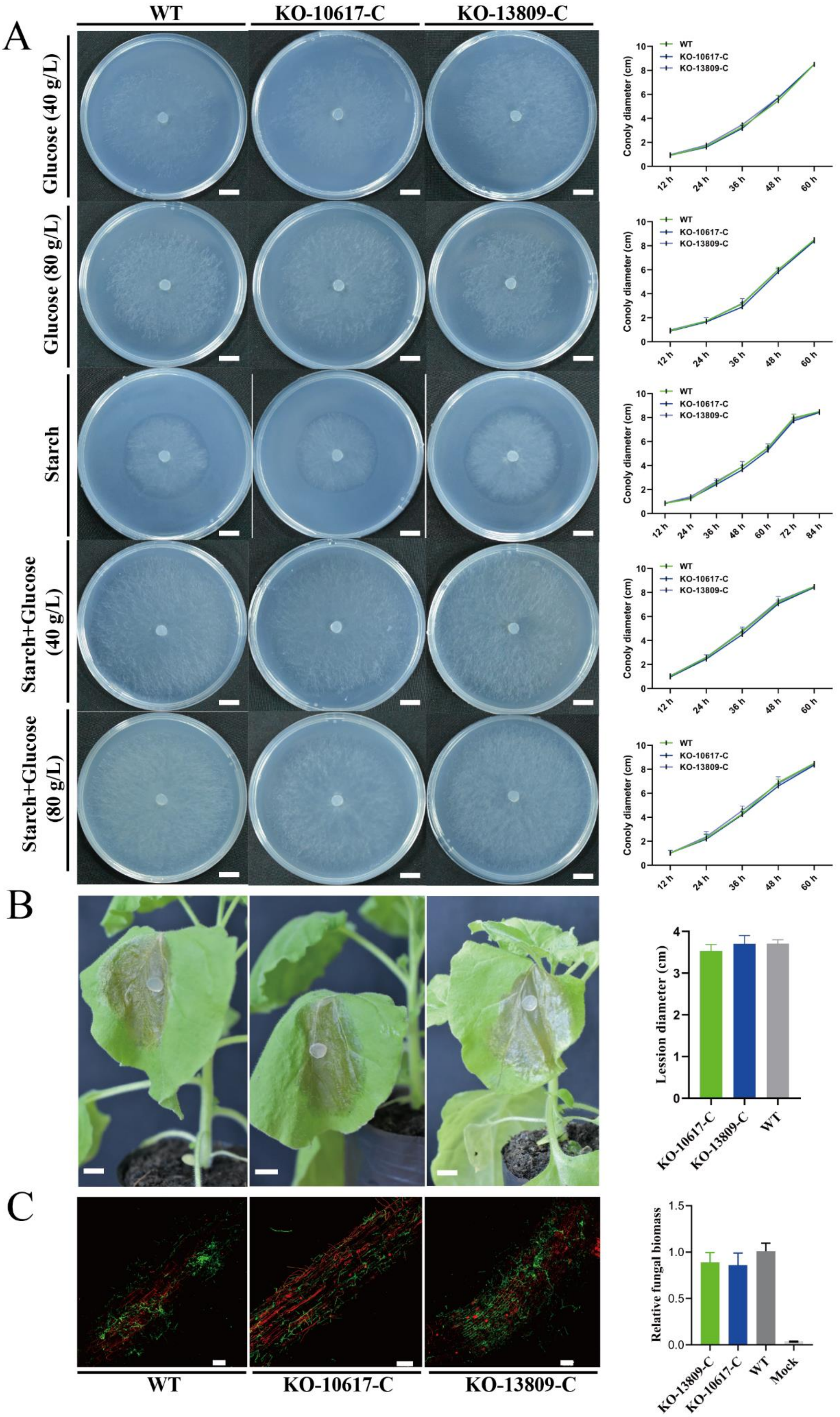

A
WT
KO-10617-C
KO-13809-C
Glucose (40 g/L)
Glucose (80 g/L)
Starch
Starch+Glucose (40 g/L)
Starch+Glucose (80 g/L)
Conoly diameter (cm)
12 h
24 h
36 h
48 h
60 h
72 h
84 h
B
Lession diameter (cm)
C
Relative fungal biomass
Mock

Fig. S9 Colony morphologies, virulence, and colonization capacity assay of KO-10617 and KO-13809 mutant complementary transformants.

(A) Colony morphologies of KO-10617 and KO-13809 complementary transformants and wild-type strain (WT) at 20 °C for 2 days on the glucose plate (40 g/L and 80 g/L), the starch plate, and starch media with glucose (40 g/L and 80 g/L). Scale bar, 1 cm. (B) The virulence of the KO-10617 and KO-13809 complementary transformants, and wild-type were assayed on the detached living *N. benthamiana* leaves at 2 dpi. (C) The colonization capacity assay of complementary transformant on the wheat host. Confocal microscopy images showing wheat roots inoculated with the *S. sclerotiorum*-WT strain, KO-10617-C and KO-13809-C at 12 dpi. Hyphae of *S. sclerotiorum* were stained with WGA, and wheat plant cell-wall apoplastic space was stained with PI. Scale bars, 100 μm. *S. sclerotiorum* biomass in roots of wheat inoculated with the KO-10617-C and KO-13809-C strains measured by qPCR using total DNA extracted at 12 dpi. *S. sclerotiorum* DNA was calculated using the threshold cycle (C_t) method, normalized to the wheat *TaEF-1a* gene, and expressed relative to that in roots inoculated with WT strain. Wheat plant roots 12 days post-inoculated with water as Mocks. Error bars indicate SD; n = 3 biological replicates.
